# Photophysical, thermal and imaging studies on vancomycin functional branched poly(*N*-isopropyl acrylamide) of differing degrees of branching containing nile red for detection of Gram-positive bacteria†

**DOI:** 10.1039/d4tb01544d

**Published:** 2024-10-18

**Authors:** Thomas Swift, Richard Hoskins, Mariya Kalinichenko, Maria Katsikogianni, Marc Daigneault, Stephen Rimmer

**Affiliations:** a School of Chemistry and Biosciences, University of Bradford BD7 1DP UK t.swift@bradford.ac.uk s.rimmer@bradford.ac.uk; b University of Sheffield S3 7HF UK

## Abstract

Highly branched poly(*N*-isopropyl acrylamide) additives chain end functionalised with vancomycin have been designed to agglutinate and report on targetted Gram-positive strains of bacteria (*S. aureus*). These branched systems selectively desolvate with temperature or binding interactions depending on their chain architecture. We have prepared samples with three different degrees of branching which have incorporated Nile red acrylate as a low concentration of co-monomer to report upon their solution properties. A linear analogue polymer functionalised with vancomycin along the chain instead of the termini is presented as a control which does not bind to targeted bacteria. These samples were analysed by diffusion NMR spectrometry (DOSY), calorimetry, fluorescence lifetime measurements, optical microscopy and scanning electron microscopy to gain a full understanding of their solution properties. The branched polymers are shown conclusively to have a core–shell structure, where the chain ends are expressed from the desolvated globule even above the lower critical solution temperature – as demonstrated by NMR measurements. The level of desolvation is critically dependent on the degree of branching, and as a result we have found intermediate structures provide optimal body temperature bacterial sensing as a consequence of the Nile red reporting dye.

## Abstract 2

Highly branched poly(*N*-isopropyl acrylamide) additives chain end functionalised with vancomycin have been designed to agglutinate and report on targetted Gram-positive strains of bacteria (*S. aureus*).

## Graphical abstract

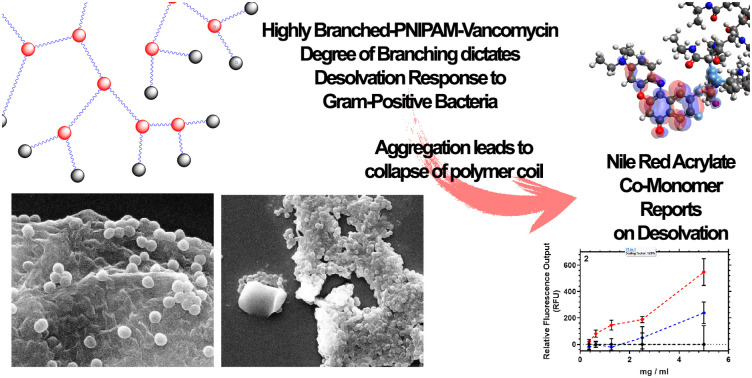

## Introduction

Rising levels of antibiotic resistance requires both new antibiotics and improved clinical diagnostics.^1^ One of the major recent breakthroughs has been the development of microfluidic chips to separate strains that are then genotyped *via in situ* fluorescence hybridization (FISH) to rapidly stratify different strains.^2^ In 2024 commercial spinouts of this technology designed to target urine infections were awarded the £8 million longitude prize, a recognition of the scale of international challenge antibiotic resistance present – and further innovations are needed to sample antimicrobial resistance in other healthcare areas and across the environment. Most current rapid diagnostics for the detection of pathogens are based on either enzyme reactions that generate colour or various formats of gene sequencing techniques.^3^ Matrix assisted laser desorption ionisation-time of flight mass spectrometry also shows promise for laboratory based identification of resistant strains.^4^

One of the major challenges of all techniques for pathogen testing is sampling. We have demonstrated a vancomycin functionalised highly branched poly(*N*-isopropyl acrylamide) (HB-PNIPAM) additive‡‡On a molar ratio, evidence suggests approx. 10% of the HB-PNIPAM chain ends are drug functionalised. The remainder will be residual imidazole RAFT (although most has been cleaved) or carboxylic acid moieties. that has been incorporated into both polyurethane foams (wound dressings)^5^ and crosslinked hydrogels (contact lenses).^6^ In the latter it has been shown to successfully sample bacteria *in vivo* from animal trials with excellent diagnostic efficacy and toxicological safety results.^7^ The effect is so pronounced these polymers are capable of extracting bacteria from a biofilm and have proposed potential in woundcare management as a direct additive.^8^ This property arises from the fact the additives are highly branched synthetic polymers with multiple end groups that bind to bacterial surfaces and desolvate on binding.^9^ Linear polymers with equivalent chemical functionality do not demonstrate equivilent desolvation responses to targeted pathogens.^9^ This suggests the additive's properties are a unique consequence of the branched polymer architecture impacting the solution properties (polmer conformation as a response to relative levels of solvation) of the material.

The unique role of branched polymers to behave independently of linear analoges are a known, but not well understood, phenomenon. Recently Moloney *et al.* demonstrated that chain extension of hyperbranched doxorubicin conjugated poly(*N*-(2-hydroxypropyl)methacrylamide) materials had completely altered toxicological impact *in vivo* despite equivalent drug loading (9 to 11%) and particle size (12–17 nm *via* DLS reported).^10^ A whole range of branched and dendritic polymer architectures are proposed as future functional nanomaterials for therapeutic delivery.^11^ A variety of novel synthetic strategies have been produced to target different chain architectures, and have been shown to impact on biopharmaceutical results.^12^ However the impact of varying chain architecture is less clear on fundamental solution properties (*i.e.* polymer conformation) and how this will vary as a result of complex architectural reconfiguration. This is further complicated by evidence that even polymers with no turbidity (*i.e.* insufficient particle formation to scatter light *via* the Tindall effect) have incomplete solubility due to sub-microscopic microgel interactions.^13^ One suggested theory is that for branched polymers end groups are pushed to the polymer surface – and thus remain more accessible for chemical interactions.^14^

There is an inherent dispersiy of products from synthetic radical reaction processes. This is most commonly associated with variations in resultant molecular weight, due to the competing kinetics of termination and propagation reactions, but it is also apparent from diversity in branching. Almost all synthetic polymers produced *via* radical reactions will result in some degree of branching, due to potential chain radical backbiting reactions, although this is often reduced by the use of chain transfer agents.^15^ However despite this variability in the synthetic precision, studies of ‘performative’ linear polymers against systems with inbuilt and regular branching segments show clear and distinct solution properties.^16^

There has been some debate about the definition of when a branched polymer gains particulate (material) like properties. Results studying star polymers have shown that crowded environments of highly branched systems exhibit properties similar to molecular rings (*i.e.* molecules that contain no free chain ends) in the molten state.^17^ Star polymers are distinct from other forms of branching though in that they exhibit one highly crowded centre of mass, whilst other systems exhibit multiple branch points throughout the chain distribution. A review of the literature indicates the difference between what is regarded as a highly branched polymer as opposed to a crosslinked structure appears to be linked to apparent solubility, or rather at what point the branching excludes significant solvent penetration. Soft, water soluble microgel dispersions have historically been characterised by a combination of light scattering and viscometric techniques.^18^

**Scheme 1:**
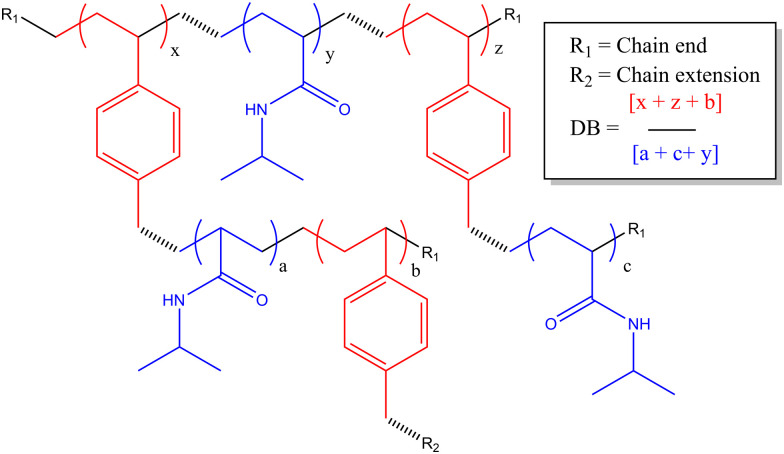
Chemical structure of HB-PNIPAM-VAN polymer backbone.

In this study we are focused on vancomycin functional branched polymers produced *via* self condensing vinyl polymerisation (SCVP). This is a method proposed in the 1990s to produce dendritic-like materials with numerous reactive groups for branching.^19^ Since then it has been applied in many different scenarios to produce a range of polymers with distinct topologies and resultant solution properties.^20^ In summary, the incorporation of ‘transmers’ (reactive multi-functional monomers) into the reaction feed results in regular branching points throughout the polymer chain. For this study we have employed a reversible addition–fragmentation chain transfer (RAFT) comonomer that produces both vinyl and dithioate functionality on either side of an aromatic ring, a system which has previously been well employed in the study of acrylate monomers.^21^ The primary monomer studied is poly(*N*-isopropylacrylamide), a thermoresponsive monomer of interest due to its lower critical solution temperature (LCST) at biologically relevant temperatures. SCVP-RAFT of PNIPAM produces broad, multimodal, high molecular weight products with low viscosity and a high density of functionalisable chain ends, as depicted in Scheme 1. Of interest it has already been shown that the LCST of these systems is impacted by the degree of branching, and that branched systems begin segmental collapse at lower temperatures than the linear polymer.^16^ This previous work has suggested SCVP-PNIPAM polymers prepared in this manner exhibit a ‘core–shell’ type structures – with varying levels of desolvation through the polymer coil.^16^ Furthermore use of the ‘spectrophotometric ruler’ to study the physical separation between fluorescent co-monomers have shown that monomers within the branched structure are affected by the chain end structure, with the polymer swelling or desolvating in response to both temperature and charge density.^22^ This was further interrogated by chain extending the outer segments of the polymer and showing segmental compartmentalisation between the ‘deeper’ side chain groups and the ‘outer’ side chains near the extremities of the branched polymer topology.^23^

HB-PNIPAM-VAN bind and respond to Gram positive species as polymer segments close to the end groups loose water and pass through a segmental coil-to-globule transition. Vancomycin binds to terminal Ala–Ala sequences in the cell wall of Gram-positive species and we showed that HB-PNIPAM-VAN also binds to the Ala–Ala motif. We also used a solvatochromic dye, Nile red, as a probe to show that the polymer responded (increase in fluorescence intensity on binding) to binding to *Staphylococcus* (*S*.) *aureus* by desolvation were as an equivalent linear polymer did not.^23^ In further work it was shown that similar polymers with Nile red covalently attached to polymer segments provided direct increases in fluorescence intensity on binding to either *S. aureus* and *S. epidermis*, and the magnitude of the increase was related to the amount of bacteria.^23^

**Scheme 2:**
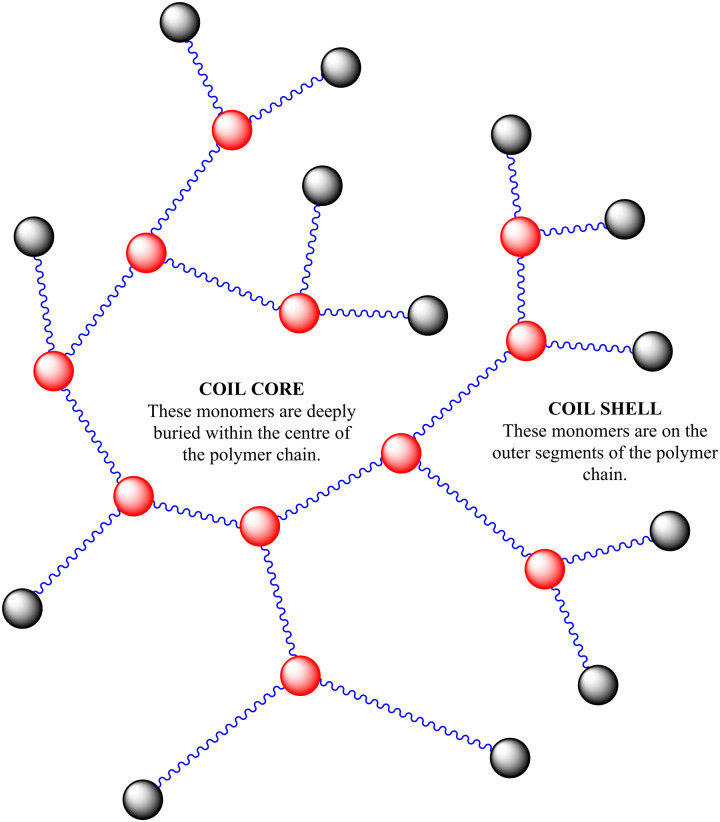
Polymer structure of highly branched polymers produced *via* self-condensing reaction mechanism.

In previous work we had not examined the effects of changing the HB-PNIPAM-VAN degree of branching (DB) on molar mass distribution,^24^ lower critical solution temperature^16^ and bacterial adhesion.^5^ There is a clear body of evidence that the chain architecture of these materials fundamentally affects their solvency in way that both positively and negatively impacts binding affinity to targets. In this work we have prepared three HB-PNIPAM-VAN polymers containing covalently attached Nile red with different DB. We previously showed that changing the DB influences the stimulus-responsive properties of the highly branched polymers with imidazole or carboxylic acid end,^22^*N*-vinyl pyrrolidone^25^ or peptide end groups.^22,26^ However, the effects of DB on HB-PNIPAM that bind and respond to bacteria have not been reported. Here we show the effects of changing DB on photophysical behaviour in different media and in the presence of *S. aureus*. We also further characterise the complex material structure of these additives, aiming to further disclose the link between complex polymer architectures (described in Scheme 2) and their functional activity.

We are particularly interested in developing our understanding of the role of the colorimetric reporting dyes in the desolvating polymer chain. The spectral properties of Nile red are dependent of the micro-environment;^27^ both solvent polarity and to a lesser extent temperature.^16^ In order to incorporate Nile red as a direct sensor to the polymer binding system we have had to alter its structure, introducing a polymerizable acrylate group to covalently attach it onto the polymer backbone.^23^ Many variations of nile red using similar modifications have been reported in the literature and are useful in biological staining. For example modification with a benzenesulfonyl group provided dyes that were sensitive to biothiols.^28^ Similarly, “click” chemistry was used to enable *in situ* binding to DNA oligonucleotides.^29^ All modifications of the Nile red appear to induce spectroscopic changes to its emission profile, which can be predicted computationally with a good degree of accuracy.^30^ Therefore we have used time-dependant density functional theory (TD-DFT) to understand changes in the HOMO–LUMO molecular orbitals responsible for its colorimetric emission following reported methods.^31^ This manuscript shows new structural analysis of the nile red functional polymers, investigates their structural and spectral properties and evidences their biological interactions to improve our understanding of this complex biochemical phenomena that will improve their integration into future theranostic applications.

## Results and discussion

### Polymer synthesis and characterisation

Polymers 1, 2 and 3 are HB-PNIPAM-VANs containing Nile red were prepared using a previously reported method (their full synthesis is collated in the ESI†). In this work the polymers had different degrees of branching (DB defined as stated in Table 1) which result in varying chain end functionalities. The amount of Nile red (NR) in the reaction feed was maintained low (0.2–0.4% wt/wt) to ensure it acted solely as a reporter on PNIPAM solution properties. Characterisation data is reported in Table 1 and size exclusion chromatograms as well as further non-parametric analysis of the molar mass distributions are provided in ESI.† Additionally we provide polymer 4 for comparison, a vancomycin functional linear P(NIPAM-*co*-Nile red) polymer whose physical material properties are further investigated here but has already been partially reported.^23^

**Table 1:** Characterisation of HB-PNIPAM-VAN and linear polymer control

Sample	NIPAM:1 (feed)	*M*_n_ (kg mol^−1^)	*M*_w_ (kg mol^−1^)	*M*_z_ (kg mol^−1^)	*Đ*	*α* a	DB^b^	B-ratio^c^	*R*_H_ d (nm)	Van^e^ (mg mL^−1^)	Van.^f^ (mass %)
1	15:1	770	1530	2210	1.98	0.16	0.107	0.0060	4.1	0.52	32.5
2	25:1	783	1754	2714	2.24	0.18	0.065	0.035	2.4	0.42	21.8
3	45:1	538	1265	2379	2.35	0.25	0.044	0.023	3.6	0.35	23.2
4	Linear	2201	3257	4278	1.47	0.51	—	—	4.2	0.33	22.2

a Mark–Houwink–Sakarada exponent extracted from SEC in methanol.

b Determined *via* [^1^H] NMR. Degree of branching, DB = (*T* + *D*)/(*T* + *D* + *L*), where *T* = terminal unit, *D* = branch point, *L* = linear unit.

c Branching ratio in parenthesis = [aryl unit]/[repeat unit].

d Size comes from DOSY sizing of largest polymer ^1^H integral peak at 298 K in D_2_O – with peak *D* value converted to hydrodynamic radii using Stokes Einstein equation.

e Vanc. content determined by ELISA, shows mg mL^−1^ of Vanc. (per mg of polymer).

f Vancomycin mass % content determined by TGA, as % mass loss Vanc. (450–700 °C) compared to mass loss HB-PNIPAM (200–450 °C).

**Fig. 1:**
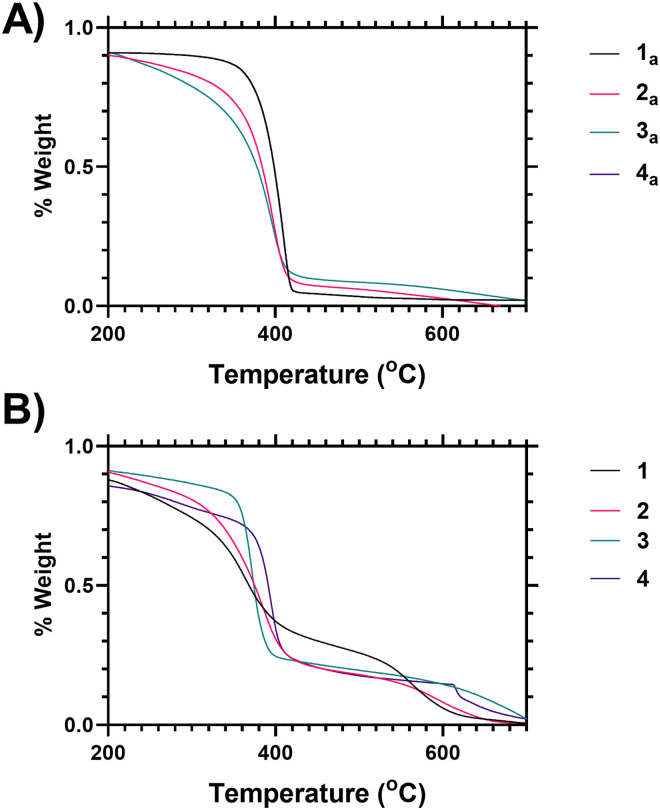
(A) Thermogravimetric analysis (TGA) mass loss profiles for polymers 1–4 (B) TGA analysis for precursor polymers 1_a_–4_a_ (unmodified pyrrole/acid chain ended products of SCVP RAFT synthesis process prior to chain end modification).

Variability in both the degree of branching and the end group functionalisation were demonstrated *via* thermogravimetric analysis as the polymers undergo degradation at elevated temperatures. Following loss of associated moisture at 50–100 °C the main decomposition event of PNIPAM occurs between 370–420 °C as shown in Fig. 1A, and analysis of the derivative mass loss of an unfunctionalized linear PNIPAM (4 precursor) polymer indicates this occurs over a narrower temperature range than the branched polymers (Fig. 1B). Thermal degradation is broadened towards lower temperature with increased branching (see ESI† for further explanation). Deconvolution of the data derivatives into distinct temperature ranges (see ESI†) shows that 38% of the linear PNIPAM degradation occurs around 410 °C, whilst only 20% occurs in the range 360–370 °C. Conversely for the highest branched polymer (1) only 12% of the degradation occurs at 410 °C with 40 occurring between 360–370 °C range. Polymers 2 and 3 have intermediate behaviour respectively. This shifting distribution of thermal stability matches the incorporation of the benzyl ring branching group, the changing architectural properties of the highly branched materials, and an increased proportional density of imidazole and acid chain ends. We hypothesize that the earlier onset of thermal degradation is due to these chain ends. The data indicates the suitability of TGA as a method of comparing varying polymer architecture.

Fig. 1 shows analysis of the end group modification, using thermogravimetric analysis (TGA), carried out by comparison of vancomycin-functional polymers to the precursor polymers (*i.e.* those synthesised *via* SCVP-RAFT polymerisation to produce a mixture of imidazole–acid chain ends). Looking at the vancomycin functionalised polymers it was found that incorporation of Vancomycin introduced an additional decomposition event at 580–650 °C (Fig. 1B), and thus TGA can also be used to indicate end group grafting efficiency (see ESI† Section 1.7 for further analysis).

The vancomycin loading at chain ends is also examined using a sandwich–enzyme-linked immunosorbent assay (ELISA) (full details are disclosed in the ESI†). The ELISA provided independent quantification of vancomycin loading and indicates an apparent slightly higher loading of vancomycin than the TGA (results are presented as concentration [mg mL^−1^] rather than mass %). All polymers had significant vancomycin attachment.

### Analysis of polymer diffusion

In solution the polymers exhibit a measurable LCST behaviour, which occurs following a coil to globule transition (*T*_c–g_) of the polymer chains. This was examined *via* both the cloud point (turbidity), by determining the enthalpy of desolvation and changes to the ^1^H NMR spectra and diffusion. First we present the diffusion analysis as this presented several novel observations.

**Fig. 2:**
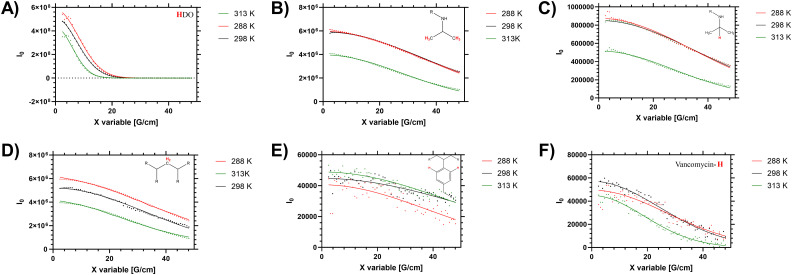
Stejskal–Tanner plots of high resolution DOSY (48 gradient decay steps) of HB-PNIPAM-Vanc (polymer 2) showing varying diffusions of different components of branching architecture in D_2_O at 288, 298 and 313 K. These represent peaks at (A) 4.69 ppm (H_2_O residue in solvent), (B) 1.1 ppm (–C**H**_**3**_ in PNIPAM isopropyl side chain), (C) 3.8 ppm (–C**H**–(CH_3_)_2_ in PNIPAM isopropyl side chain), (D) 1.5 ppm (CH_2_ in polymer backbone), (E) 7.4 ppm (aromatic peaks from benzyl branching group), (F) 6.47 ppm (vancomycin aromatic-H peak from functional chain ends).

Diffusion NMR measurements were used to probe the dispersity of coil size of the sample. Different proton environments across the disperse branching polymer architecture were seen to give different peak diffusion values – both in water (shown in Fig. 2 and Table 2) and organic solvents (see ESI†). Of particular interest was variation of *D* between the monomer (side chain) protons, the benzyl branching sub-units, and the chain end (vancomycin) moieties. In linear (narrow dispersity) polymers these moieties have equivalent diffusion values (as shown for polymer 4 in ESI† Fig. S5). The variation in the average peak diffusion values reflect the structural heterogeneity of the branched polymers. Depending on the degree of desolvation it is possible that portions of the polymer would become ‘invisible’ to the solution-state NMR.^13^ However for this system the observed DB and B values (shown in Table 1) follow closely the reaction feed trend, and there is no gross change in observed DB when changing solvents (*i.e.* from water to DMSO) indicating the majority of the polymer is ‘visible’. Thus, the assumption that the *D* values below the LCST are representative of the entire polymer distribution will be used but we acknowledge the potential for deviation depending on the level of desolvation (particularly at higher temperatures).

Intensity and diffusion of ^1^H signals from HB-PNIPAM-Vanc in D_2_O

**Table:** 

Temp. (K)	Peak intensity (integrated to solvent (4.7 ppm) peak)					
	1.1 ppm	1.5 ppm	3.8 ppm	4.7 ppm	6.5 ppm	7.4 ppm
288	7.91	3.69	1.39	100	0.038	0.150
298	7.84	3.65	1.38	100	0.039	0.148
313	6.88	3.12	1.21	100	0.038	0.145
Δ^a^	13%	15%	12%	—	0%	3%

a Δ is the % reduction of integrated proton signal for broad ^1^H peak as sample is heated from 288 to 313 K. This is distinct from initial intensity of proton signal in Stejskal–Tanner plots (Fig. 2) which only shows proton intensity at exactly peak centre (precise ppm matching) and does not account for peak broadening.

**Table:** 

Temp. (K)	Peak diffusion (× 10^−10^ M^2^ S^−1^)					
	1.1 ppm	1.5 ppm	3.8 ppm	4.7 ppm	6.5 ppm	7.4 ppm
288	0.72	0.91	0.78	14.1	1.27	0.40
298	0.69	0.85	0.73	16.1	1.62	0.39
313	1.17	1.34	1.22	26.4	2.61	0.41

When studying linear polymers, it is possible to comment on apparent size dispersities from DOSY data (*i.e*. provide a *Đ* approximately equivalent to *M*_w_/*M*_n_ values)^32^ but this process is more complex for samples with multiple branching points and multiple chain ends. Here it is possible to identify specified [^1^H] environments that represent polymer size distribution (number average) based on the main monomer sidechain, the benzyl branching unit and multiple different chain end distributions. For low intensity (broad) ^1^H signals (such as the benzl protons) there are clear shimming distortions that disrupt the data (see Fig. 2E) – but the fitting profiles of the Stejskal–Tanner plots shown in Fig. 2 indicate a reasonable fit to the produced decay curve despite inherent noise of the underlying data below the LCST. Finally the supernatant (residual H_2_O in the D_2_O solution) was probed to determine an accurate in sample viscosity, thus providing accurate hydrodynamic radii *via* use of the Stokes Einstein equation, as we have previously demonstrated.^24^ In the branched polymer these peaks show varying peak average *D* values depending on their distribution through the branched coil.

Elevated temperature analysis of polymer 2 show that despite the material desolvating at elevated temperature, some portion of the material remains visible to the spectrometer even above *T*_c–g_ (*i.e.* 40 °C, 313 K). We note that the PNIPAM monomer side chain protons (Fig. 2B and C) were reduced as the material was heated across the LCST, whilst the (low intensity) branching (benzyl aromatic) and chain end vancomycin (Fig. 2E and F) were not reduced significantly. Conversely in the linear polymer the vancomycin (loaded along the side chain) signal was reduced to a much greater degree (see ESI† Section 1.4). This is an important measurement that supports several hypotheses on the behaviour of these branched materials. Firstly, the maintenance of peak intensity of the vancomycin proton signal compared to the PNIPAM backbone indicates that the chain ends remain expressed to the outside of the branched polymer as the core desolvates. Secondly, the observation that the signal of the semi-precipitated materials is retained at all follows observation that the vancomycin provides some degree of colloidal stability; making the cloud point not just temperature sensitive but also responsive to factors such as concentration, ionic strength, heating rate and equilibriation time. We note that equivilent polymers with structure intermediate to 1 and 2 have previously been reported to exhibit cloud points in excess of 55 °C in PBS.^33^ Thus there is clearly some degree of colloidal stability provided by the vancomycin, which retains a degree of solvation of at least ‘segments’ of the polymer chain, retaining visibility to the NMR spectrometer.

**Fig. 3:**
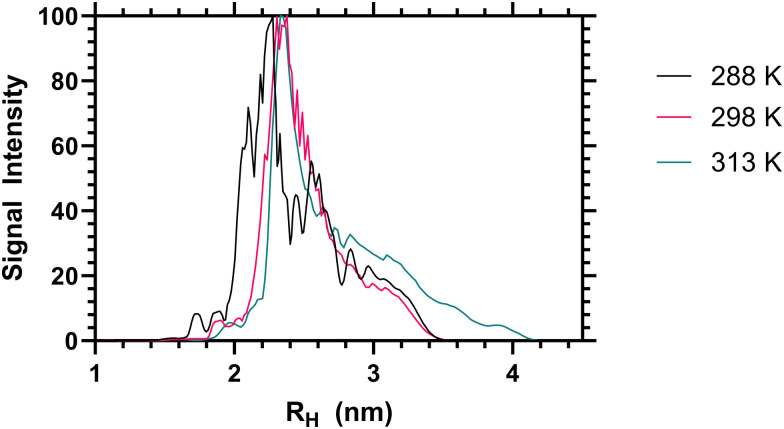
Apparent hydrodynamic radii of polymer 2 from high resolution DOSY NMR analysis, as a 1 mg mL^−1^ solution in D_2_O at 288, 298 and 313 K.

When the sum diffusion distribution of the polymer [^1^H] signal was plotted above and below the LCST the changing shape of the polymer chains can be determined. At 288, 298 and 313 K polymer 2 exhibited changing peak diffusion values of 6.6, 7.2 and 0.17 × 10^−11^ M^2^ S^−1^ respectively (see ESI† for full datasets). However, as the solution viscosity varied with both sample temperature and polymer rheology, the peak apparant hydrodynamic radii of the dissolved chains is not substantively altered as shown in Fig. 3. Here it can be seen that, the primary ^1^H signal peak (arising primarily from the polymer side chain protons) remains at ≈2.4 nm both above and below the LCST. However, as the signal intensity of these side chains was reduced (as shown in Table 2) it is likely that the signal is biased towards the more solvated ‘segments’.

### Solution properties

The behaviour at *T*_c–g_ is influenced by binding of the vancomycin end groups to the AlaAla peptide; the target for vancomycin in Gram-positive bacteria. Binding of the polymers to these targets is indicative of their potential to bind to pathogens. Table 3 shows the thermoresponsive behaviour obtained by turbimetry, *T*_c–g_ values obtained by calorimetry in PBS alone and in the presence of AlaAla. Data showing the LCST in pure water of these polymers, and the precursor (non functionalised materials) are shown in the ESI† (Section 1.3).

**Table 3:** Cloud point and *T*_c–g_ measured alone and in the presence of AlaAla (1 mg mL^−1^, PBS)

	Cloud Pt^a^ (°C)*	Cloud Pt + AlaAla^a^ (°C)*	*T*_c–g_ b (°C)	*T*_c–g_ + AlaAla^b^ (°C)	Δ*q*^c^ (%)
1	16.5	14.6	21.5	19.7	1
2	16.3	14.8	28.9	19.3	27
3	17.1	14.7	29.8	30.1	33
4	39	37	34.0	33.9	17

a *T*_c–g_ measured by calorimetry.

b Cloud points measured by turbimetry.

c Δ*q* shows %reduction in binding enthalpy (mJ) of transition enthalpy in presence/absence of the Ala–Ala peptide.

**Fig. 4:**
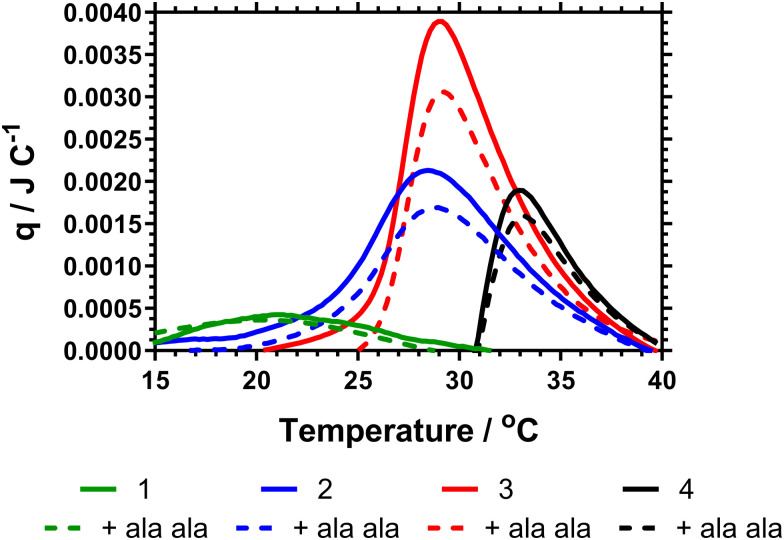
Micro-DSC thermogram for HB-PNIPAM-Vanc in absence (solid lines) and presence (dashed lines) of d-Ala–d-Ala peptide in PBS. Contains vanc. functionalised polymers with 1 (DB 0.107 (green)), 2 (DB 0.038 (blue)), 3 (0.021 (red)) and 4 (linear polymer (black)).

The data Table 3 showed that increased DB led to a decrease in both the cloud point and *T*_c–g_ as measured by calorimetry. This is expected as shown in the previous literature, branching introduces hydrophobic aryl functionality and also increases segmental crowding of the system; both of which affect solution solubility. The cloud point data also indicated a decrease in the cloud point on adding Ala–Ala to the polymers. However, Fig. 4 provides the calorimetric data and shows that addition of Ala–Ala did not affect *T*_c–g_. Polymer 1 (DB = 0.107) contained the largest amount of vancomycin but although the *T*_c–g_ was observed by microDSC the data were not affected by adding Ala–Ala. The observation of a much reduced endotherm for this polymer confirmed the earlier reported data where we showed that high DB in HB-PNIPAM with carboxylic acid end groups produced polymers in which the core segments did not become solvated. In this polymer, the endotherm was derived only from desolvation of the end segments. For polymers 2 and 3 (DB = 0.065 and 0.044) the data in Fig. 4 showed that there was a decrease in the heat required to progress the coil-to globule transition on binding to Ala–Ala and Table 2 shows that the endotherms are reduced by ≈0.03 J on binding Ala–Ala. Polymer 4 reduces to a lesser extent. The data indicated that for polymers 2 and 3 the Ala–Ala bound to the vancomycin groups and that the adjacent chain end segments became desolvated on binding; that is lower amounts of heat were required at the *T*_c–g_ to further desolvate the polymer chains due to entropic LCST rearrangements.

The observation that desolvation of polymer 1 was not reduced on binding support previous studies showing that this polymer was in a semi-collapsed state at all temperatures. This was originally demonstrated by Plenderleith *et al.* and observed using a non-attached Nile red probe.^16^ Less branched polymers only approach this point at higher temperatures after undergoing the LCST transition.

### Photophysical behaviour

**Fig. 5:**
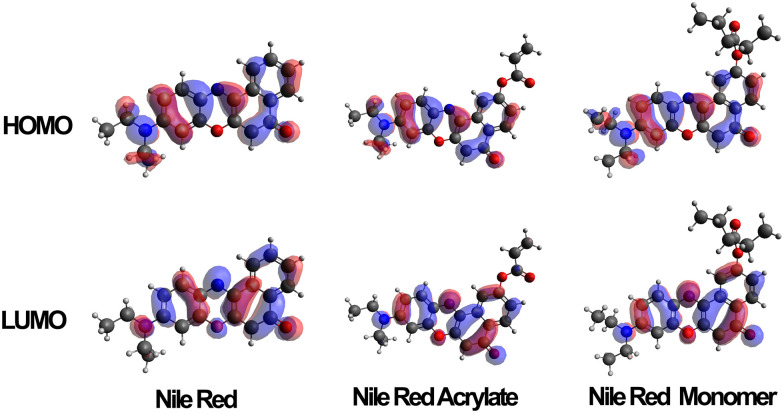
HOMO and LUMO orbitals of Nile red, Nile red acrylate and Nile red monomer when calculated in an aqueous solvent conditions (*via* CPCM).

Several variations of Nile red were modelled to predict the electron distribution in the frontier molecular orbitals. The variations were carried out on both unmodified Nile red, the polymerizable intermediate Nile red acrylate, a monomeric unit of the dye along a carbon–carbon polymer chain and finally a trimer where the unit was complemented by two adjacent NIPAM side chains. Once these structures had been optimised in the gas state molecular orbital calculations were carried out using a TD-DFT/B3LYP/DEF2-TZVP calculation. Typical distribution of HOMO and LUMO are shown in Fig. 5. The HOMO contribution of Nile red is spread evenly across the molecule, whilst the LUMO shifts it away from the diethylamino group, focusing the electronic contribution around the four six-membered rings that form the core of the dye. When the MO calculations were carried out on the unpolymerized and then monomeric acrylate functional dye this situation was retained, indicating a clear modelling prediction that even when bound to the polymer chains the polymerised Nile red dye will have equivalent spectroscopic electronic properties to the free dye in solutions. Furthermore its responsiveness to external solution polarity (*i.e.* change of S_1_, S_2_ and S_3_ excited state transitions) using implicit solvation models (conductor like polarisable continuum model) is equivalent, as shown in the ESI.†

**Fig. 6:**
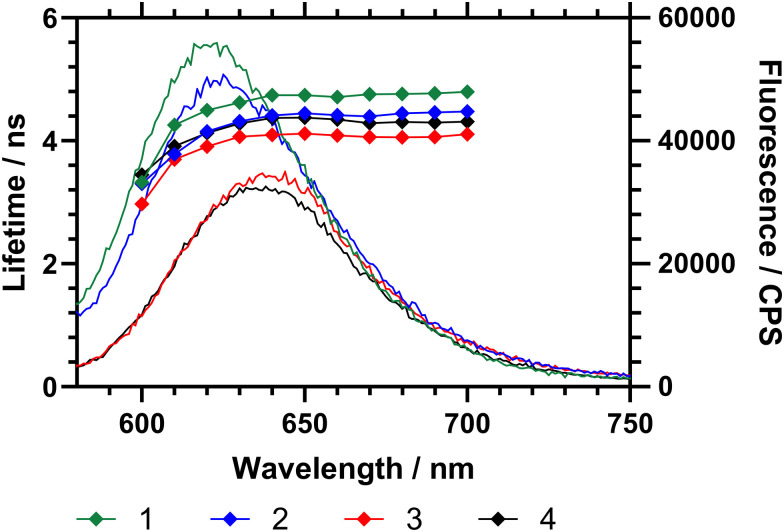
Fluorescence emission spectra (15 °C) of HB-P(NIPAM-*co*-NR)-VAN polymers with emission spectra (left axis) and emission wavelength specific excited state lifetime (right axis). *λ*_ex_ = 570 nm. Polymer 1 (DB 0.107) is black (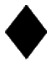); 2 (DB 0.065) is red (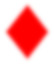); 3 (DB 0.044) is blue (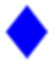).

The emission spectrum of Nile red is dependent on the polarity of its microenvironment. Desolvation (coil-to-globule) conformational change of a surrounding polymer affects the local environment of the dye and produces a change of micropolarity.^16^ To examine the effects of DB the fluorescence emission (wavelength and intensity) and fluorescence decay (excited state lifetime) of the attached Nile red were considered for the three polymers with different DB. First the emission of Nile red labelled polymers (1–3) in water at 15 °C (below *T*_c–g_ for each polymer) was studied (Fig. 6). These data showed that, as the branching of the polymers decreases, the maximum of the emission shifts to a higher wavelength. Fig. 6 also showed that the excited state lifetime of the dye (*τ*) increases with DB. Within a sample the average fluorescence lifetime changes slightly, decreasing sharply below 610 nm. Across the range 620–700 nm the lifetime averages of polymers 1–4 were *τ*_1_ = 4.71 ± 0.09 ns, *τ*_2_ = 4.39 ± 0.10 ns, *τ*_3_ = 4.06 ± 0.06 and *τ*_4_ = 4.30 ± 0.07 ns respectively. The NR comonomer in the polymers is randomly distributed across the polymer, and there is a dispersity in the environment, it is reasonable to conclude that the fluorescence lifetime increases as the environment becomes increasingly hydrophobic.

Both the maximum emission wavelength and the lifetime data indicated that below the *T*_c–g_ the average polarity of the polymer chain decreased (indicating reduced solvation of the chains) with branching. The fluorescence spectra were then obtained in ultrapure water at temperatures between 10 to 45 °C, as shown in Table 4 and Fig. 6. Both the maximum emission wavelength and the lifetime data indicated that below the *T*_c–g_ the average polarity of the polymer chain decreased (indicating reduced solvation of the chains). Table 4 also shows the fluorescence lifetime and correlation time (rotational diffusion of the dye as determined *via* time resolved polarised anisotropy) in both water and PBS. This analysis shows the amount of quenching the Nile red is subjected to changes with the different degrees of polymer collapse. Polymers 1 and 2 provided substantially more shielding than polymer 3 and 4 below the LCST. The anisotropy meausrements do not report quenching but the segmental mobility (correlation time (*τ*_C_) – which relates to rotational diffusion) of the dye during its excited state. Here 2 shows the lowest *τ*_C_ in both water and PBS.

Fluorescence intensity, excited state lifetime and polarised anisotropy of polymers 1–4 in H_2_O and PBS (1 mg mL^−1^)^a^

**Table:** 

H_2_O					
Sample	*I* (20 °C)	*I* (40 °C)	*τ* (ns)^b^ (20 °C)	*τ* (ns)^b^ (40 °C)	*τ*_C_ (ns)^b^ (20 °C)
1	44 300	47 000	4.69 ± 0.16	4.69 ± 0.16	3.29 ± 0.53
2	29 400	55 200	4.44 ± 0.15	4.38 ± 0.17	2.26 ± 0.16
3	29 300	46 500	4.03 ± 0. 06	4.23 ± 0.06	8.55 ± 2.73
4	16 540	72 220	4.29 ± 0.16	4.40 ± 0.13	12.45 ± 3.12

a Fluorescence emission intensity, lifetime and anisotropy recorded at *λ*_ex_ 570 nm, *λ*_em_ 640 nm. Fluorescent intensities and lifetimes at other temperatures are reported in the ESI.

b Raw data is shown in the ESI.

**Table:** 

PBS					
Sample	*I* (20 °C)	*I* (40 °C)	*τ* (ns)^b^ (20 °C)	*τ* (ns)^b^ (40 °C)	*τ*_C_ (ns)^b^ (20 °C)
1	39 700	39 400	4.62 ± 0.18	4.54 ± 0.15	4.46 ± 1.02
2	28 700	32 300	4.46 ± 0.06	4.32 ± 0.06	1.62 ± 1.83
3	13 800	18 500	4.07 ± 0.15	4.05 ± 0.15	2.73 ± 4.36
4	14 810	16 900	2.45 ± 0.15	3.37 ± 0.16	7.34 ± 2.05

**Fig. 7:**
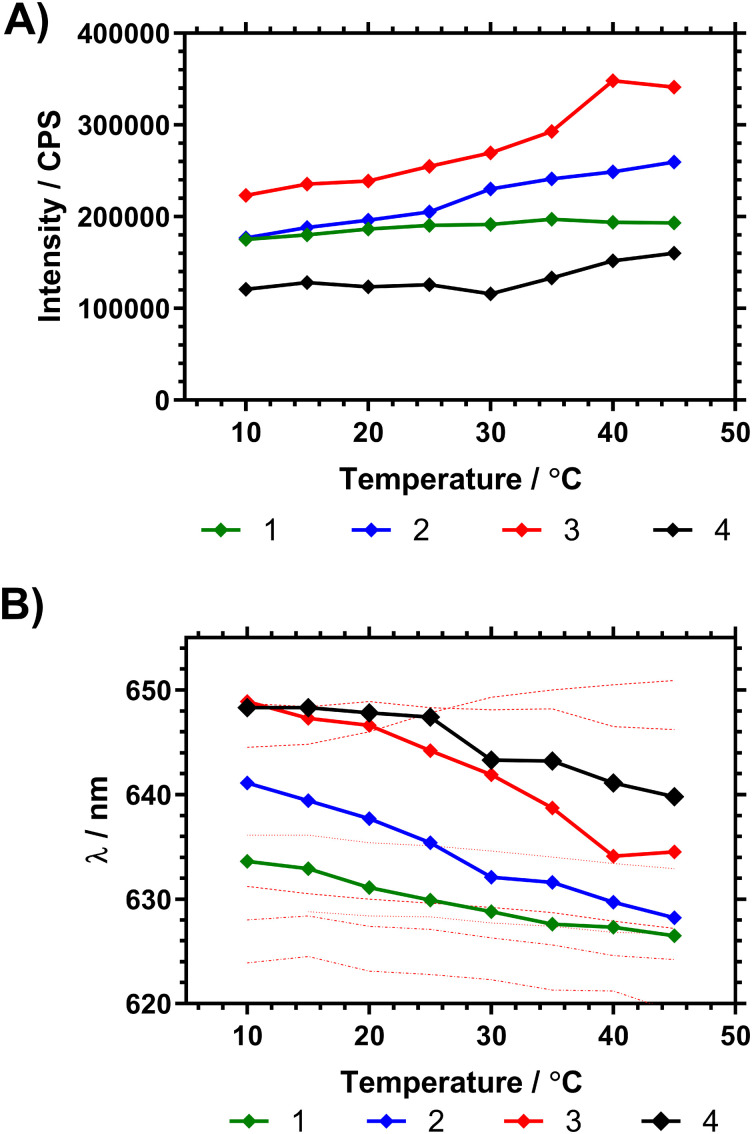
(A) Shift in peak NR emission intensity of dilute (1 mg mL^−1^ H_2_O) polymer solutions following *λ*_ex_ 580 nm. (B) Shift in peak fluorescence emission wavelength (average mean of distribution) following *λ*_ex_ 580 nm, with temperature of pyrrole chain end polymers compared to NR solvent shifts (solvents listed from max. to min. λ_em_ at 15 °C using data from Plenderleith *et al.*^16^ ethylene glycol, glycerol, methanol, ethanol, DMSO, butanol, propan-2-ol).

Fig. 7 shows the fluorescence spectra in ultrapure water between 10 and 45 °C, demonstrating changes in fluorescence intensity and wavelength as the polymers progressed through the *T*_c–g_. These studies were also repeated on precursor polymers (materials with a mixture of RAFT (imidazole) and acid chain ends) and in PBS – all of which is shown in the ESI.† The data showed that the presence of the vancomycin chain ends had a large impact on the responsiveness of the Nile red covalently bound to the inside of the polymer coil. Comparing emission of polymers in H_2_O and PBS solutions however led to wavelength shifts that varied with the degree of branching (Δ*λ*1: ≈ 1 nm, 2: ≈ 4 nm, 3: ≈ 4 nm, 4: ≈ 8 nm).

**Fig. 8:**
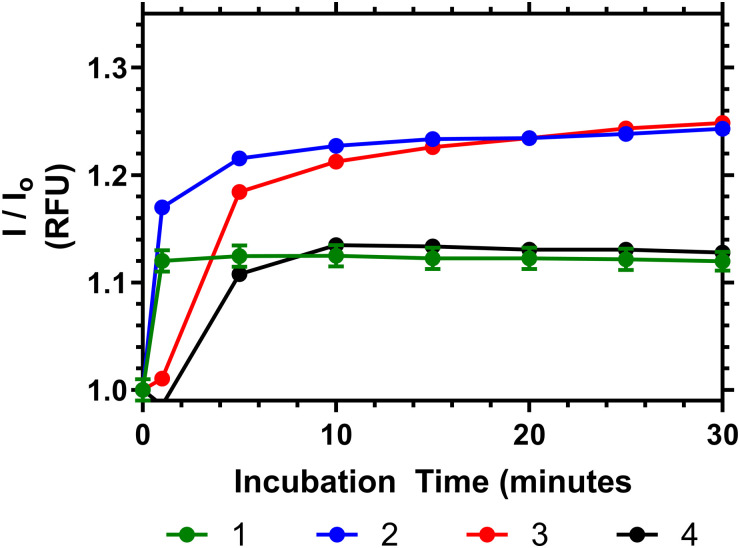
Relative fluorescence output of following addition of Ala–Ala peptide to solutions of 1–4 at 35 °C (5 mg mL^−1^), compared to polymers initial emission once equilibriated to temperature in PBS.

To demonstrate the potential of these polymers for bacterial interactions studies were carried out to show their thermal response to d-Ala–d-Ala peptides. This was done at multiple temperatures (see ESI†) and the one closest to body temperature (35 °C) is shown in Fig. 8. Here it can be seen that, despite the observation that the polymers have already partially collapsed, a substantial emission increase was observed in all samples over 10 to 20 minutes after Ala–Ala addition. At lower temperatures (10 to 25 °C) however polymers 3 and 4 did not demonstrate increases in emission intensity; indicating that these polymers were not responsive to the Ala–Ala group unless already partially desolvated (as shown in the Fig. S17, ESI†).

Analysis of the data in Fig. 8 shows that Polymer 1 and 2 respond the most rapidly to addition of the Ala–Ala peptide compared to polymers 3 and 4, whilst polymers 2 and 3 show the greatest % change of relative fluorescence intensity. Thus Polymer 2 appears to be an optimal formulation with the fastest and most intense response. In previous work we have shown that polymers similar to 1 have substantially desolvated cores. Thus, although this polymer responded rapidly to the binding only the more solvated outer segments responded and the degree of increase in intensity was much less than for polymers 2 and 3. Comparing polymers 2 and 3 increased branching appears to have increased the rate of response and more work is needed to fully rationalise this behaviour.

Binding of the polymers to these targets is indicative of their potential to bind to pathogens. However we know that polymer 4 does not provide a colorimetric response to *S. aureus* or *S. epidermis.*^9,23^ This work shows that there is a temperature dependent enthalpic (Fig. 4) and colorimetric (Fig. 8) response to the interaction between vancomycin and Ala–Ala, so binding remains viable, but the linear polymer does not lead to microbe aggregation to the same extent compared to the branched polymers.

### Interaction of HB-PNIPAM-VAN with *Staphylococcus aureus*

**Fig. 9:**
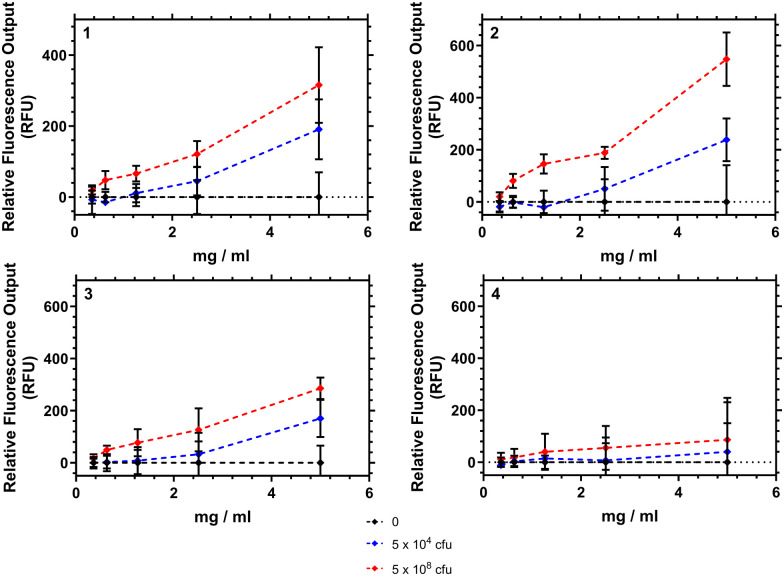
Fluorescence emission intensity plotted against polymer–bacteria (*S. aureus*) concentration. Sample recorded after 2 hour incubation at 37 °C, then measurement read at 32 °C at emission 639 nm. Ties indicate significance (ANOVA, general linear model, *post hoc* Tukey) between samples 1–3 (*p < 0.05, **p < 0.01, ***p < 0.001). Error bars show variation over 5 repeat measurements for each sample.

Fig. 9 shows how the fluorescence emission intensity of polymers 1–4 in response to bacteria could be measured using a typical laboratory fluorescent plate reader. Here the polymers were exposed to different concentrations of bacteria (10^4^ or 10^8^ colony forming units (CFU) mL^−1^) and the emission intensity recorded at 639 nm. Polymers 1–3 showed an increase in fluorescence intensity as the amount of bacteria in suspension increased. The differences at the concentrations of bacteria were significant (*p* < 0.05) when the polymer concentration was 5 mg mL^−1^. Polymer 4 however demonstrated no significant increase in emission.

We note that the interaction of these polymers are purely aggregrative, and that polymer 2 when tested showed no overnight bacteriacidal activity (see ESI†). The emission increase was tested against 2 strains of *S. aureus* (Oxford and S-235) and was confirmed not to have any interaction with a negative control *P. aeruginosa*. A matt button assay (study of biofilm formation) from mixing different concentrations of polymers 1–4 with different concentrations of bacteria found a concentration of polymer in excess of 2.5 mg mL^−1^ was necessary to prevent formation of *S. aureus* buttons within well plates. Confocal analysis of aggregates showed that as polymer concentration was increased the bacteria were pulled into particles which increased in size in a roughly linear fashion.

### Scanning electron microscopy imaging of polymer–bacteria interactions

**Fig. 10:**
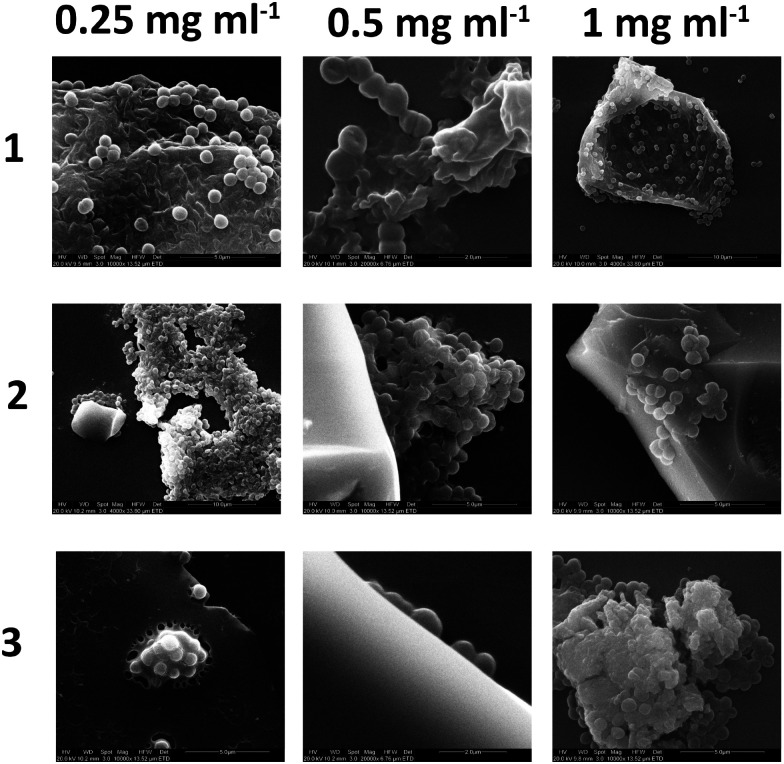
SEM images of combined 10^8^ cfu *S. aureus* + HB-PNIPAM-Vanc solutions dried on a glass plate.

Bacteria, after culture and resuspension in PBS, were allowed to interact with the three branched polymers, at various concentrations (0.25–1 mg mL^−1^), for 4 hours and were subsequenlty allowed to attach to glass slides for a further 4 hours before been fixed, dehydrated, gold sputtered and examined under SEM. Fig. 10 shows that the increase of polymer concentration enhances polymer–bacteria interactions and polymer aggregation, especially for polymer 2. This is in agreement with the fluorescence intensity data presented in Fig. 9. From the data presented in the ESI† (Fig. S26), polymer only and bacteria only SEM images confirm that polymer aggregation occurs only in the presence of Gram-positive bacteria and not in the presence of Gram-negative ones or without the presence of bacteria. When bacteria attach on the glass surface in the absence of polymer, for up to 4 hours, no aggregation was observed. This confirms that polymer–Gram-positive bacteria interactions are the ones that drive polymer aggregation in solution.

Fig. 10 shows that the shape and size of the aggregates varies with both polymer structure and relative concentration to the bacterial suspension. In isolation the polymers dry on the glass slide to produce residues of amorphous matter and salt crystals (from the PBS) as shown in the ESI.† In the presence of bacteria there is clear interactions of polymers 1–3 with the *S. aureus* as it appears to agglutinate the bacteria with high degrees of exopolymer present that was not visible when the bacteria was cultured alone (see ESI†). As the concentration of polymer was increased from 0.25 to 1 mg mL^−1^ increased amounts of polymer film formation appeared, although this was observed to a certain extent in all samples. Additional negative control studies with Gram-negative *E. coli* and *P. aeruginosa* did not show any bacterial aggregation in the presence of the polymer, and images are presented in the ESI.†

### Experimental

#### Materials

HB-PNIPAM-VAN were synthesised using previously reported methods.^5,23^ Full details are provided in ESI.†

#### NMR

Measurements in deuterated solvents were carried out on either a Bruker AC250, AC500 or Avance Neo, operated at 250, 500 or 600 MHz respectively. ^1^H DOSY measurements using a stebpgp1s sequence were collected using a DiffBB probe, using Bruker Topspin (v 4.3.0) and analysed within the Dynamic Centre Module.

#### Determination of LCST

Micro differential scanning calorimetry (MicroDSC) of polymers and Ala–Ala peptide was conducted using a VP-DSC microcalorimeter. The transition temperature (LCST) of polymer preparations was defined as the temperature corresponding to the peak of the thermogram. The samples were prepared in deionised water at 1 mg cm^−3^, degassed using ThermoVac. The LCST was determined over a range of temperatures from 10–40 °C at a heating rate of 0.5 °C min^−1^. The vancomycin-modified polymer concentration was 10 mg cm^−3^. Turbimetry was used to measure the cloud point of polymers with a Cary 300 bio UV-visible spectrophotometer. The cloud point was determined at 550 nm over a range of temperatures from 10–70 °C. For this the samples were prepared in deionised water at a concentration of 1 mg mL^−1^ and a heating rate of 1 °C min^−1^. The onset temperature was defined as the first stage of onset of turbidity. In the case of vancomycin-derivatised polymers (1–4) the concentration was 10 mg cm^−3^.

#### Ab initio modelling

All modelling was carried out using the software ORCA.^34^

#### Fluorescence emission spectroscopy

Fluorescence emission measurements were obtained using a Horiba Scientific Fluoromax-4 spectrometer. Sample excitation was carried out at 580 nm with a slit width of 2 nm, and the emission spectrum was observed from 585 to 800 nm with a slit width of 2 nm. Each spectra was repeated 10 times to obtain the average emission profile. The emission wavelength of broad peaks was calculated by fitting the emission profile to a gaussian distribution and calculating the mean wavelength in Graphpad Prism 6. Exicted state lifetime and anisotropy measurements were carried out using a HORIBA TCSPC Fluorolog accessory with nanoLED excitation sources with emission profiles measured across a 200 nanosecond range with 4010 channels.

#### Scanning electron microscopy

A reference strain of *S. aureus* (‘Oxford’ NCTC 6571) was used as representative Gram-positive bacteria, while *E. coli* (NCTC 12923) and *Pseudomonas* (*P*.) *aeruginosa* (SOM-1) were used as representative Gram-negative bacteria. Polymer–bacteria interactions were examined using Fluorescence emission spectroscopy, as described above, and scanning electron micorcopy (SEM) using a FEI Quanta 400 E-SEM instrument under vacuum conditions. The details of the bacterial cultures and sample preparation are presented in the ESI.†

## Conclusions

This work supports previously established evidence that the complex architecture of the highly branched polymer additives studied in this paper is necessary for the desired biological interaction as a theranostic additive. In previous works it was established these polymers had a ‘core–shell’ structure below the LCST,^16^ with a desolvated core and more solvated segments at the periphery. Using DOSY NMR the work presented provides further evidence for the core shell structure.

Highly branched polymers 1–3 show broad desolvation ranges as different segments of the polymer architecture rearrange with temperature. Polymer 1 is already extensively desolvated even below its apparant LCST, and thus the Δ*q* for *T*_c–g_ in presence and absence of Ala–Ala binding targets is small compared to other polymers. Linear polymer 4, despite having an approximately equivalent loading of vancomycin to the polymer 3, also has a Δ*q* that is reduced. Polymers 2 and 3 show the greatest change in enthalpy of desolvation on Ala–Ala addition, and thus the largest macromolecular rearrangement to binding. These ‘partially’ desolvated systems are thus a useful tool for detecting bacteria.

The covalently incorporated Nile red dye has been shown to be extremely sensitive to the conformational changes of the polymer. It shows that polymers 1–4 all respond to Ala–Ala addition within a timescale of minutes, it re-confirms that branched structures are necessary for bacterial aggregation. This paper shows that a balance between branching and desolvation potential is necessary to optimise the fluorescence response of the reporter system to both bacteria targets (Ala–Ala) and samples from microbiological culture. These branched polymer–bacterial aggregates were then studied by scanning electron microscopy to provide our most in depth look to date at the aggregation process – as emergent particles of specified bacteria cluster around the precipitated polymer.

## Author contributions

Conceptualisation: T. S. + S. R. Resources: T. S. + S. R.; data curation: T. S., R. H., M. D. Formal analysis; R. H., M. K., M. D., T. S. Investigation: R. H., M. K., M. D., T. S. Methodology: T. S., M. K. Project administration: T. S. + S. R. Resources; T. S. + S. R. Supervision: S. R., T. S., M. K. Writing – original draft: T. S. Writing – review and editing: T. S., S. R., M. K.

## Data availability

The data supporting this article have been included as part of the ESI.†

## Conflicts of interest

There are no conflicts to declare.

## Supplementary Material

TB-012-D4TB01544D-s001

TB-012-D4TB01544D-s002
