# Supplementary material for: Photophysical, thermal and imaging studies on vancomycin functional branched poly(N-isopropyl acrylamide) of differing degrees of branching containing nile red for detection of Gram-positive bacteria
Source: J Mater Chem B. 2024 Oct 18;12(46):11996–2006. doi: 10.1039/d4tb01544d (PMC11503812; doi:10.1039/d4tb01544d)
Supplement: TB-012-D4TB01544D-s002 [file TB-012-D4TB01544D-s002.pdf]

Supplementary Information (SI) for Journal of Materials Chemistry B.
This journal is © The Royal Society of Chemistry 2024

Photophysical, Thermal and Imaging Studies on Vancomycin Functional Branched Poly(*N*-isopropyl acrylamide) of Differing Degrees of Branching Containing Nile Red for Detection of Gram-Positive Bacteria

Electronic Supporting Information

Table of Contents

1.1 Raw Materials and Analytical Methods **2**

1.2 Polymer Synthesis **6**

1.3 Polymers for Comparison **10**

1.4 Nuclear Magnetic Resonance Analysis **13**

1.5 FTIR Analysis **20**

1.6 Polymer Molar Mass Analysis and Vancomycin Loading **21**

1.7 Polymer Cloud point + LCST Studies **23**

1.8 Thermal Degradation of Polymer Chain Ends **25**

1.9 Additional Diffusion NMR Analysis **27**

1.10 Ab Initio Modelling **30**

1.11 Fluorescence of PNIPAM NR **33**

1.12 PNIPAM + Bacteria **37**

2. ESI References **44**

1.1 Raw Materials and Analytical Methods

All materials were used as supplied unless otherwise stated. Diethyl amino phenol (97% purity), sodium nitrite (>97% purity), 1,6, dihydroxy naphthalene (99% purity), acryloyl chloride (97% purity), triethylamine, N-isopropyl acrylamide (97% purity), N-hydroxy succinimide (98% purity), dicyclohexyl carbodiimide (98% purity), vancomycin hydrochloride hydrate, polymyxin acylase, dichloromethane (HPLC grade) and ethyl acetate (HPLC grade) were obtained from Sigma Aldrich. N-isopropyl acrylamide was recrystallized from hexane/toluene. 4,4'-Azobiscyano valeric acid (98% purity) was obtained from Alfa Aeser. Vinyl benzoic acid (98% purity) and polymyxin B sulphate were obtained from Fluka. RAFT agent 4-Vinylbenzyl- pyrrolecarbodithioate was synthesised and purified according to a previously reported method. Dioxane (Analar grade), diethyl ether (anhydrous), dimethyl formamide (Analar grade), ethanol (Analar grade), acetone (Analar grade) and hydrochloric acid (35% wt/wt) were obtained from VWR. Mouse monoclonal and rabbit polyclonal antibodies to vancomycin and goat antirabbit IgG conjugated to horse radish peroxidase (HRP) were obtained from Abcam Plc (Cambridge, U.K.) and diluted for use. Deuterated NMR solvents were purchased from Cambridge Isotope Laboratories Inc. All materials containing nile red acrylate co-monomers were stored in darkened cupboards to avoid photobleaching on storage.

Synthesis of 4-vinylbenzyl pyrrolecarbodithioate (RAFT Agent)

This is a modification of a method previously published[1–3]. A 250 ml sealed reaction vessel was purged with nitrogen before sodium hydroxide (2.98 g, 74.53 mmol, (in a mineral oil dispersion)) and dimethylformamide (DMF, 80 ml) were added. Pyrrole (5.00 g, 74.53 mmol) dissolved in DMF (10 ml) was added dropwise to the rapidly stirring suspension over a period of 30 minutes to obtain a yellow foam, before the product was cooled to 0°C in an ice bath. Carbon disulphide (5.68 g, 4.50 ml, 74.53 mmol) dissolved in DMF (10 ml) was added dropwise for 10 minutes using a pressure equalising funnel to produce a dark red solution that was allowed to warm back to room temperature with continuous stirring. Finally the solution was cooled back to 0°C as 4-vinylbenzyl chloride (11.37 g, 10.50 ml, 74.53 mmol) in DMF (10 ml) was added dropwise over a period of 20 minutes using a pressure equalising funnel. The solution was stirred for a further 16 hours at room temperature before diethyl ether (80 ml) and distilled water (80 ml) were added to phase separate the products. The organic layer, containing 4-Vinylbenzyl-Pyrrolecarbodithioate, was recovered whereas the aqueous layer consisting of residue was extracted with diethyl ether (160 ml) three times. The extracts were dried over magnesium sulphate and filtered. The solvent was removed by rotary evaporation to yield a brown oil. The product was purified by flash chromatography using 100% hexane as an eluent (Rf value of the product (bright yellow oil) was 0.180). The solvent was removed by rotary evaporation and the final product was stored at -18°C under an atmosphere of N_2. ^{1}H NMR ($CDCl_3$, ca. 5% CD_3OD, 250 MHz): δ/ppm: 7.7 (2H, m, N-CH=, pyrrole); 7.4 (4H, s, C6H4-); 6.7 (1H, dd, Jcis = 10.9Hz, Jtrans = 17.6Hz, vinyl); 6.3 (2H, m, =CH-, pyrrole); 5.8 (1H, d, Jtrans = 17.6Hz, vinyl) ; 5.3 (1H, d, Jcis = 10.9Hz, vinyl) ; 4.6 (2H, s, Ar-CH2-S-). ^{13}C NMR ($CDCl_3$, ca. 5% CD_3OD, RT, 62.5 MHz): δ/ppm: 199.5 (1C, S-C=S); 137.5 (1C, aromatic C-CH2-S); 136.5(1C, Ar-CH=CH2, vinyl); 134.12 (1C, aromatic C-CH=CH2); 129.8 (2C, aromatic); 126.74 (2C, aromatic); 120.85 (2C, -N-CH=CH, pyrrole); 114.55 (1C, -CH=CH2 vinyl); 114.42 (2C, -N-CH=CH, pyrrole); 41.68 (1C, S-CH2-Ar). Elemental Analysis, % found (expected): Carbon 65.30% (64.83%); Hydrogen 5.00% (5.05%); Nitrogen 5.30% (5.40%); Sulphur 22.4% (24.72%).

Synthesis of Nile red acrylate

This is a modification of a method previously published.[4] 3-Diethylaminophenol (5 g) was dissolved in a mixture of concentrated HCl (11 ml) and water (6 ml) and cooled on ice. A solution of sodium nitrite (2.1 g) in water (35 ml) was added dropwise over 1 hour and the resulting slurry stirred on ice for a further 2 hours. The crude product was dissolved in boiling ethanol and recrystallized with diethyl ether to yield a yellow/orange solid (3.7g, 50% yield) identified as 5-Diethylamino-2-nitrosophenol hydrochloride (mass spectrometry found m/z = 195, expected 195.) 5-Diethylamino-2-nitrosophenol hydrochloride (1.5 g) and 1,6-dihydroxynaphthalene (1.05 g) were dissolved in DMF (180ml) and refluxed for 7 hours. The solvent was removed and the residue purified by silica gel column chromatography (petroleum spirit:ethyl acetate 20%-100%) yielding 0.52 g (20%) of a dark blue solid identified as hydroxy nile red. ^{1}H NMR in DMSO d6 : δ = 10.4 (1H, s), 7.95 (1H, d), 7.88 (1H, d), 7.6 (1H, d), 7.08 (1H, d), 6.8 (1H, d), 6.6 (1H, d), 6.15 (1H,s), 3.5 (4H, q), 1.18 (6H, t). Mass spectrometry found m/z = 335, expected 335. Hydroxy nile (0.5 g) was dissolved in dichloromethane (140 ml) and triethylamine (870 ml) and acryloyl chloride (500 ml) added. The solution was stirred at room temperature for 7 hours. Solvent was removed and the residue purified by silica gel column chloromatography (petroleum spirit:ethyl acetate (2:1) yielding 0.2g (34 %). ^{1}H NMR in DMSO d6 : δ = 8.3 (1H, d), 8.2 (1H, d), 7.6 (1H, d), 7.5 (1H, d), 6.85 (1H, d), 6.75 (1H, d), 6.6 (1H, d), 6.5 (1H, q), 6.3(1H, s), 6.2 (1H, d), 3.5 (4H, q), 1.2 (6H, t). Mass spectrometry found m/z = 389, expected 389.

^{1}H NMR spectroscopy

Oriignal ^{1}H NMR spectra were measured and recorded on Bruker AC250 and AC500 which were operated at 250 MHz and 500 MHz with $CDCl_3$, DMSO, D_2O as the solvent at 20 mg ml^{-1} concentration. ^{1}H DOSY measurements were recorded on a Bruker Avance Neo 600 MHz instrument with a DiffBB probe. For these measurements high precision field matched NMR tubes were purchased from Wilmad USA. Calibration of the gradient field strength was performed using a sample of H2O in D2O (1% v/v) doped with GdCl3 (0.1 mg) as a paramagnetic relaxation agent. The spectrometer was controlled using Bruker Topspin 4.3.0 to collect and process NMR data, with diffusion values interpreted using Bruker Dynamics Centre software.

A visual representation of the DOSY pulse sequence is shown below in Scheme ESI 1.

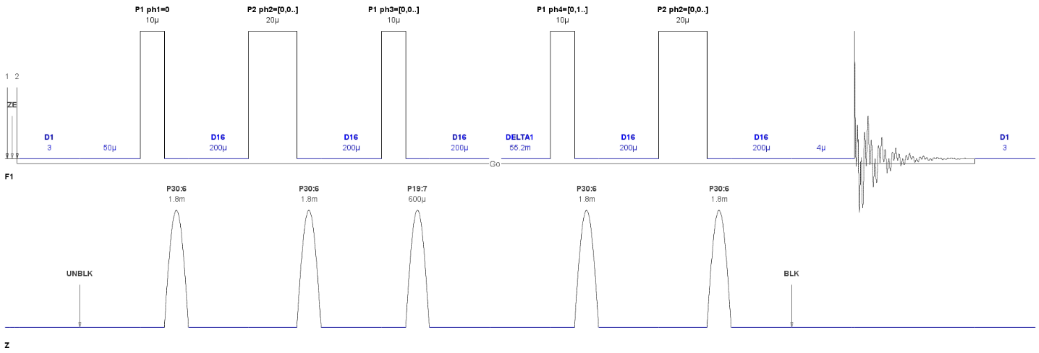

Scheme ESI 1 – NMR pulse sequence for DOSY measurements.

Fourier Transform Infrared Ramen spectroscopy (FTIR)

FTIR measurements were carried out on a Thermo Scientific Nicolet iS10 FT-IR Spectrometer. Solid samples were dried in a vacuum oven overnight before use.

Thermogravimetric Analysis (TGA)

TGA analysis was carried out on a TA Instruments Q5000 IR TGA, with < 5 mg of sample heated at a 1 °C min^{-1} heating rate. Analysis of data was carried out on a TA Universal Analysis software (V4.5.0.5).

Size Exclusion Chromatography (SEC)

This is use of methodology first published in 2017[5].Molecular weights of polymers was determined by SEC with methanol as eluent. Samples were prepared in pre-filtered methanol at 1mg/ml and injected through two Agilent PolarGel columns at a rate of 1 ml min^{-1}, maintained at a constant 30 °C. They were analysed via comparison to a universal calibration using PNIPAM standards via Agilent Refractive Index and Viscometric (Agilent 1260 Infinity detector Suite) detectors to give absolute molecular weight averages (M_n, M_w, M_n and two forms of dispersity (Đ) M_w /M_n and M_z/M_w).

Vancomycin ELISA

This utilised a methodology previously published[6]. In this method a sandwich-enzyme-linked immunosorbent assay (ELISA) was used to determine quantities of vancomycin on polymer chains. Flat bottomed ELISA 96-well plates (Micro-lon, high binding Grenier bio-one) were coated with mouse antivancomycin monoclonal antibody diluted 1/500 in carbonate buffer pH 9.6 at 4 °C overnight. After washing with PBS-tween (0.05%v/v tween) four times to remove unbound antibody, wells were blocked with 3% (w/v) bovine serum albumin (BSA) in PBS for 1 h at37 or 4 °C overnight in order to block unoccupied binding sites on the polystyrene. Excess blocking agent was removed by washing with PBS-tween four times, and then, polymer samples and controls (PBS blank, HB-PNIPAM-COOH) in 1% BSA/PBS were added to wells and incubated overnight at 4 °C. Wells were again washed four times with PBS-tween, and rabbit antivancomycin polyclonal diluted 1/3000 in1% BSA/PBS was added and incubated for 2 h at room temperature followed by washing with PBS-tween four times. Finally, goat antirabbit IgG conjugated to HRP in 1% BSA/PBS was added for 1h at room temperature. After washing, wells were developed by the addition of 3,3,5,5-tetramethylbenzidine substrate plus hydrogen peroxide (30% v/v). The reaction was stopped after 25 min by addition of 2 M H_2SO_4 and the resultant colour measured at 450 nm

Bacteria

A reference strain of *S. aureus* (‘Oxford’ NCTC 6571) was used as representative Gram-positive bacteria, while *E. coli* (NCTC 12923) and *Pseudomonas* (P.) *aeruginosa* (SOM-1) was used as representative Gram-negative bacteria. Bacteria were cultured under standard conditions on brain heart infusion (BHI) agar (Oxoid), and in BHI broth for experimental use. Following overnight incubation in broth, bacteria were washed and re-suspended in PBS.

Fluorescence Microscopy

Images were captured and processed on a Zeiss Axiovert 200M inverted fluorescence microscope using Axiovision Rel 4.6 software. To directly stain bacteria (as opposed to polymer) the samples were labelled with Alexa Fluoro 488 (NHS). The bacteria were incubated with the polymer for 8 hours after labelled with dyes to ensure they were visible by optical microscopes as clear bright spots within each panel.

Scanning Electron Microscopy

Scanning Electron Microscopy (SEM) of the bacteria-polymer interactions was using a FEI Quanta 400 E-SEM instrument under vacuum conditions. For the sample preparation, bacteria, after overnight culture in BHI broth, were centrifuged and re-suspended in PBS. 1 ml of 10^8 cfu of bacteria was incubated with 1 ml of the various polymers at different concentrations for 4 hours and subsequently samples were added on glass slides where they were allowed to attach for a further 4 hours. Samples were the fixed using 2.5% glutaraldehyde and dehydrated using increasing concentrations of ethanol. The samples were then gold sputtered using an Emitech sputter coater (Quorum Technologies, UK) and were then examined using SEM at various magnifications. For the bacteria only and polymers only samples, the bacteria in PBS were allowed to attach on the glass slides and the polymer only solutions at various concentrations were again allowed to attach on glass slides for 4 hours prior to fixation, dehydration and sputtering.

Matt Button Assay

Polymer and bacteria agglutination (matt-button formation) tests were carried out following established methods.[3,4] 40 µl each of test polymer (5 mg ml^{-1}) was incubated together with 40 µl of either PBS or bacteria suspended in PBS and ethidium bromide (5 µg cm^{-3}) at 1 $x10^4$ and 1 $x10^8$ cfu cm^{-3} at 37 °C for 18 hours in U-shaped sterile 96-well plates (Greiner). 40 µl of bacteria were also incubated with 40 µl PBS as a further control. Wells were imaged under UV light in a G:Box gel documentation system (Syngene) using Genesnap software (Syngene) and images were inverted. The experiments were repeated three times, and the same results were observed in each replicate, which matches previous observations.

1.2 Polymer Synthesis

Vancomycin functional polymers **1** - **4** were prepared via functionalisation of P(NIPAM-*co*-NR), either prepared via reversible addition-fragmentation chain transfer self condensing vinyl polymerisation (**1** - **3**) or with a 25 : 1 vinyl benzoic acid comonomer (**4**). Synthesis of these materials is a three step process, with two identifiable precursors (raw polymers and acid functionalised polymers) isolated in preparation of the final material. For identification purposes these are labelled as 1-4[a] and 1-4[b] throughout this manuscript.

Synthesis of Highly Branched Poly(*N*-isopropylacrylamide-*co*-NR) with 15 : 1 RAFT : Monomer Feed (1[a])

N-isopropylacrylamide (NIPAM) (10 g, 0.088 moles), RAFT agent (1.53 g, 0.0059 moles), ACVA (1.65 g, 0.0059 moles) and nile red acrylate (0.02 g, 0.00005 moles) were dissolved in 50 ml dioxane and added to a glass ampule which was then degassed by three freeze thaw cycles and polymerised at 60 °C for 48 hours. The polymer was purified by precipitating twice in diethyl ether giving pink solid with a yield of 94%. ^{1}H NMR (400 MHz,$CDCl_3$) (ppm): d 0.9-1.1 (6H, s, -$N(CH_3)_2$), d 1.3-1.7 (2H, m, -CH_2-CH-Ar-), d 1.8-2.5 (2H, m, -CH2-CH-CO-NH-) and (1H, m, CH_2-CH-CONH -), d 3.8 (1H, s, $(CH_3)_2$CH-), d 6.4 (H2, s, N-pyrrole-H), d 6.6-7.6 (m, -Ar-), d 7.7 (2H, s, N-pyrrole-H). FTIR: 3200 cm^{-1} (sp2 aromatic C-H), 1630 cm^{-1} (sp2 amide C=O), 1570 cm^{-1} (sp2 benzene C=C), 1540 cm^{-1} (sp3 pyrrole C-N), 1500 cm^{-1} (sp3 C-C).

Synthesis of Highly Branched Poly(*N*-isopropylacrylamide-*co*-NR) with 25 : 1 RAFT : Monomer Feed (2[a])

NIPAM (10 g, 0.088 moles), RAFT agent (0.91 g, 0.0035 moles), ACVA (0.99 g, 0.0035 moles) and nile red acrylate (0.02 g, 0.00005 moles) were dissolved in 50 ml dioxane and added to a glass ampule which was degassed by three freeze thaw cycles and polymerised at 60 °C for 48 hours. Polymer was purified by precipitating twice in diethyl ether giving pink solid with a yield of 90%. ^{1}H NMR (400 MHz,$CDCl_3$) (ppm): d 0.9-1.1 (6H, s, -$N(CH_3)_2$), d 1.3-1.7 (2H, m, - CH_2-CH-Ar-), d 1.8-2.5 (2H, m, -CH_2-CH-CO-NH-) and (1H, m, CH2-CH-CONH -), d 3.8 (1H, s, $(CH_3)_2$CH-), d 6.4 (H2, s, N-pyrrole-H), d 6.6-7.6 (m, -Ar-), d 7.7 (2H, s, N-pyrrole-H). FTIR: 3200 cm^{-1} (sp2 aromatic C-H), 1630 cm^{-1} (sp2 amide C=O), 1570 cm^{-1} (sp2 benzene C=C), 1540 cm^{-1} (sp3 pyrrole C-N), 1500 cm^{-1} (sp3 C-C).

Synthesis of Highly Branched Poly(*N*-isopropylacrylamide-*co*-NR) with 45 : 1 RAFT : Monomer Feed (3[a])

NIPAM (10 g, 0.088 moles), RAFT agent (0.5 g, 0.0019 moles), ACVA (0.54 g, 0.0019 moles) and nile red acrylate (0.02 g, 0.00005 moles) were dissolved in 50 ml dioxane and added to a glass ampule which was degassed by three freeze thaw cycles and polymerised at 60 °C for 48 hours. Polymer was purified by precipitating twice in diethyl ether giving pink solid with a yield of 97%. ^{1}H NMR (400 MHz,$CDCl_3$) (ppm): d 0.9-1.1 (6H, s, -N(CH3)2), d 1.3-1.7 (2H, m, - CH2-CH-Ar-), d 1.8-2.5 (2H, m, -CH2-CH-CO-NH-) and (1H, m, CH2-CH-CONH -), d 3.8 (1H, s, (CH3)2CH-), d 6.4 (H2, s, N-pyrrole-H), d 6.6-7.6 (m, -Ar-), d 7.7 (2H, s, N-pyrrole-H). FTIR: 3200 cm^{-1} (sp2 aromatic C-H), 1630 cm^{-1} (sp2 amide C=O), 1570 cm^{-1} (sp2 benzene C=C), 1540 cm^{-1} (sp3 pyrrole C-N), 1500 cm-1 (sp3 C-C).

Synthesis of Linear P(NIPAM-co-NR) (4[a])

NIPAM (5 g, 0.044 moles) and nile red acrylate (0.01 g, 0.000025 moles) were dissolved in dioxane (45 ml) with a small amount of DMF (5 ml) added to a glass ampule which was degassed by three freeze thaw cycles and polymerised at 60 °C for 48 hours. Polymer was purified by precipitating twice in diethyl ether giving pink solid with a yield of 90%.

Acid Modification of Highly Branched Poly(*N*-isopropylacrylamide-*co*-NR) with 15 : 1 RAFT : Monomer Feed (1[b])

Poly(*N*-isopropylacrylamide-*co*-NR) (1[a]) (5 g) was dissolved in DMF (45 ml) and heated to 65 °C in the presence of ACVA (15 g, 0.053 moles). The addition of ACVA was repeated twice at 24 hour intervals. After a total of 72 hours the polymer was precipitated in diethyl ether and purified by dissolving in ethanol and ultrafiltering three times and then recovered by rotary evaporation to remove solvent. ^{1}H NMR (400 MHz, DMSO) (ppm): d 0.9-1.1 (6H, s, -N(CH_3)$_2$), d 1.3-1.7 (2H, m, - CH_2-CH-Ar-), d 1.8-2.5 (2H, m, -CH_2-CH-CO-NH-) and (1H m, CH_2-CH-CONH-), d 3.8 (1H, s, (CH_3)$_2$CH-), d 6.4 (2H, s, N-pyrrole-H), d 6.6-7.6 (m, -Ar-), d 7.7 (2H, s, N-pyrrole-H), d 12.0 (m, COOH). FTIR: 3400 cm^{-1} (O-H), 3200 cm^{-1} (sp2 aromatic C-H), 1710 cm^{-1} (sp2 carboxylic acid C=O), 1630 cm^{-1} (sp2 amide C=O), 1570 cm^{-1} (sp2 benzene C=C), 1500 cm^{-1} (sp3 C- C)

Acid Modification of Highly Branched Poly(*N*-isopropylacrylamide-*co*-NR) with 25 : 1 RAFT : Monomer Feed (2[b])

Poly(N-isopropylacrylamide-co-NR) (2[a]) (5 g) was dissolved in DMF (45ml) and heated to 65C and ACVA (9.8g, 0.035 moles) added. The addition of ACVA was repeated twice at 24 hour intervals. After a total of 72 hours the polymer was precipitated in diethyl ether and purified by dissolving in ethanol and ultrafiltering three times and then recovered by rotary evaporation to remove solvent. ^{1}H NMR (400 MHz, DMSO) (ppm): d 0.9-1.1 (6H, s, -N(CH_3)$_2$), d 1.3-1.7 (2H, m, - CH_2-CH-Ar-), d 1.8-2.5 (2H, m, -CH_2-CH-CO-NH-) and (1H m, CH_2-CH-CONH-), d 3.8 (1H, s, (CH_3)$_2$CH-), d 6.4 (2H, s, N-pyrrole-H), d 6.6-7.6 (m, -Ar-), d 7.7 (2H, s, N-pyrrole-H), d 12.0 (m, COOH). FTIR: 3400 cm^{-1} (O-H), 3200 cm^{-1} (sp2 aromatic C-H), 1710 cm^{-1} (sp2 carboxylic acid C=O), 1630 cm^{-1} (sp2 amide C=O), 1570 cm^{-1} (sp2 benzene C=C), 1500 cm^{-1} (sp3 C- C)

Acid Modification of Highly Branched Poly(*N*-isopropylacrylamide-*co*-NR) with 45 : 1 RAFT : Monomer Feed (3[b])

Poly(*N*-isopropylacrylamide-co-NR) (3[a]) (3 g) was dissolved in DMF (45 ml) and heated to 65 °C and ACVA (3.24 g, 0.011 moles) added. The addition of ACVA was repeated twice at 24 hour intervals. After a total of 72 hours the polymer was precipitated in diethyl ether and purified by dissolving in ethanol and ultrafiltering three times and then recovered by rotary evaporation to remove solvent. ^{1}H NMR (400 MHz, DMSO) (ppm): d 0.9-1.1 (6H, s, -N(CH_3)$_2$), d 1.3-1.7 (2H, m, - CH_2-CH-Ar-), d 1.8-2.5 (2H, m, -CH2-CH-CO-NH-) and (1H m, CH2-CH-CONH-), d 3.8 (1H, s, (CH3)2CH-), d 6.4 (2H, s, N-pyrrole-H), d 6.6-7.6 (m, -Ar-), d 7.7 (2H, s, N-pyrrole-H), d 12.0 (m, COOH). FTIR: 3400 cm^{-1} (O-H), 3200 cm^{-1} (sp2 aromatic C-H), 1710 cm^{-1} (sp2 carboxylic acid C=O), 1630 cm^{-1} (sp2 amide C=O), 1570 cm^{-1} (sp2 benzene C=C), 1500 cm^{-1} (sp3 C- C).

Synthesis of P(NIPAM-co-NR-co-VBA) (4[b])

NIPAM (6 g, 0.055 moles) and ACVA (0.15 g, 0.0005 moles), vinyl benzoic acid (0.30 g, 0.0020 moles), and nile red acrylate (0.012 g 0.000039 moles were dissolved in a mixture of dioxane (37 ml) and

DMF (7.5 ml) and added to a glass ampule and degassed by three freeze thaw cycles and polymerised 60 °C for 48 hours. The polymer was purified by precipitating twice in diethyl ether, yield was 92%. ^{1}H NMR (400 MHz, DMSO) (ppm): d 0.9-1.1 (6H, s, -N(CH_3)$_2$), d 1.3-1.7 (2H, m, - CH_2-CH-Ar-), d 1.8-2.2 (2H, m, -CH2-CH-CO-NH- and 1H, m, CH_2-CH-COONH-), d 3.9 (1H, s, (CH_3)$_2$CH-), d 6.6-7.6 (br m, -Ar-), d 12.2 (br m COOH). FTIR: 3300 cm-1 (O-H), 1710 cm^{-1} (sp2 carboxylic acid C=O), 1630 cm^{-1} (sp2 amide C=O), 1570 cm^{-1} (sp2 benzene C=C), 1500 cm^{-1} (sp3 C-C)

Vancomycin Modification of Highly Branched Poly(*N*-isopropylacrylamide-*co*-NR) with 15 : 1 RAFT : Monomer Feed (1)

Acid modified polymer (1^b) (1.5g) was dissolved in DMF (20ml) and N-hydroxy succinimide (0.4225g, 0.0037moles) and DCC (0.75g, 0.0036moles) in DMF (5ml) added and the solution stirred overnight at room temperature. The mixture was filtered and the polymer precipitated into diethyl ether. The solid was dissolved in ethanol and concentrated by ultra-filtration and then rotary evaporated. Succinimide derivatized polymer (300 mg) was dissolved in water (10 ml) over ice. A solution of vancomycin (135 mg, 0.00009 moles) in water (5 ml) and phosphate buffered sa-line pH 8.5 (5 ml) was added and the pH of the solution adjusted to 9.5 with NaOH (1 M). The solution was stirred overnight and purified by ultrafiltration and then freeze dried. ^{1}H NMR (500 MHz, D_2O) (ppm): d 0.9-1.1 (6H, s, -N(CH_3)$_2$), d 1.3-1.7 (2H, m, - CH_2-CH-Ar-), d 1.8-2.2 (2H, m, -CH_2-CH-CO-NH-) and (1H, m, CH_2-CH-CONH-), d 3.9 (1H, s, (CH_3)$_2$CH-), d 6.4 (H_2, s, N-pyrrole-H), d 6.5-7.6 (m, -Ar-), d 7.7 (2H, s, N-pyrrole-H). FTIR: 3300-3500 cm^{-1} (phenol O-H), 3200 cm^{-1} (sp2 aromatic C-H), 1630 cm^{-1} (sp2 amide C=O), 1570 cm^{-1} (sp2 benzene C=C), 1500 cm^{-1} (sp3 C-C)

Vancomycin Modification of Highly Branched Poly(*N*-isopropylacrylamide-*co*-NR) with 25 : 1 RAFT : Monomer Feed (**2**)

Acid modified polymer (2^b) (1.5 g) was dissolved in DMF (20 ml) and N-hydroxy succinimide (0.255 g, 0.0022 moles) and DCC (0.453 g, 0.0021 moles) in DMF (5 ml) added and the solution stirred overnight at room temperature. The mixture was filtered and the polymer precipitated into diethyl ether. The solid was dissolved in ethanol and con-centrated by ultra-filtration and then rotary evaporated. Succinimide derivatized polymer (300 mg) was dissolved in water (10 ml) over ice. A solution of vancomycin (135 mg, 0.00009 moles) in water (5 ml) and phosphate buffered sa-line pH 8.5 (5 ml) was added and the pH of the solution adjusted to 9.5 with NaOH (1 M). The solution was stirred overnight and purified by ultrafiltration and then freeze dried. ^{1}H NMR (500 MHz, D_2O) (ppm): d 0.9-1.1 (6H, s, -N(CH3)2), d 1.3-1.7 (2H, m, - CH_2-CH-Ar-), d 1.8-2.2 (2H, m, -CH_2-CH-CO-NH-) and (1H, m, CH_2-CH-CONH-), d 3.9 (1H, s, (CH3)2CH-), d 6.4 (H_2, s, N-pyrrole-H), d 6.5-7.6 (m, -Ar-), d 7.7 (2H, s, N-pyrrole-H). FTIR: 3300-3500 cm^{-1} (phenol O-H), 3200 cm^{-1} (sp2 aromatic C-H), 1630 cm^{-1} (sp2 amide C=O), 1570 cm^{-1} (sp2 benzene C=C), 1500 cm^{-1} (sp3 C-C)

Vancomycin Modification of Highly Branched Poly(*N*-isopropylacrylamide-*co*-NR) with 45 : 1 RAFT : Monomer Feed (3)

Acid modified polymer (3^b) (1 g) was dissolved in DMF (15 ml) and *N*-hydroxy succinimide (0.094 g, 0.0008 moles) and DCC (0.166g, 0.0008moles) in DMF (5ml) added and the solution stirred overnight at room temperature. The mixture was filtered and the polymer precipitated into diethyl ether. The solid was dissolved in ethanol and concentrated by ultra-filtration and then rotary evaporated.

Succinimide derivatized polymer (300 mg) was dissolved in water (10 ml) over ice. A solution of vancomycin (135 mg, 0.00009 moles) in water (5 ml) and phosphate buffered sa-line pH 8.5 (5 ml) was added and the pH of the solution adjusted to 9.5 with NaOH (1 M). The solution was stirred overnight and purified by ultrafiltration and then freeze dried. ^{1}H NMR (500 MHz, D_2O) (ppm): d 0.9-1.1 (6H, s, -$N(CH_3)_2$), d 1.3-1.7 (2H, m, - CH_2-CH-Ar-), d 1.8-2.2 (2H, m, -CH2-CH-CO-NH-) and (1H, m, CH2-CH-CONH-), d 3.9 (1H, s, (CH3)2CH-), d 6.4 (H2, s, N-pyrrole-H), d 6.5-7.6 (m, -Ar-), d 7.7 (2H, s, N-pyrrole-H). FTIR: 3300-3500 cm^{-1} (phenol O-H), 3200 cm^{-1} (sp2 aromatic C-H), 1630 cm^{-1} (sp2 amide C=O), 1570 cm^{-1} (sp2 benzene C=C), 1500 cm^{-1} (sp3 C-C).

Vancomycin Modification of P(NIPAM-co-NR-co-VBA) (4)

Poly(*N*-isopropylacrylamide-*co*-NR-*co*-vinyl benzoic acid (4[b]) (1.5 g) was dissolved in DMF (20 ml) and *N*-hydroxy succinimide (0.255 g, 0.0022 moles) and DCC (0.453g, 0.0021moles) in DMF (5ml) added and the solution stirred overnight at room temperature. The mixture was filtered and the polymer precipitated into diethyl ether. The solid was dissolved in ethanol and concentrated by ultra-filtration and then rotary evaporated. Succinimide derivatized polymer (300 mg) was dissolved in water (10 ml) over ice. A solution of vancomycin (135 mg, 0.00009 moles) in water (5 ml) and phosphate buffered saline pH 8.5 (5 ml) was added and the pH of the solution adjusted to 9.5 with NaOH (1 M). The solution was stirred overnight and purified by ultrafiltration and then freeze dried. ^{1}H NMR (500 MHz, D_2O) (ppm): d 0.9-1.1 (6H, s, -$N(CH_3)_2$), d 1.3-1.7 (2H, m, - CH2-CH-Ar-), d 1.8-2.2 (2H, m, -CH2-CH-CO-NH- and 1H, m, CH2-CH-COONH-), d 3.9 (1H, s, $(CH_3)_2$CH-), d 6.5-7.6 (m, -Ar-). FTIR: 3300-3500 cm^{-1} (phenol O-H), 3200 cm^{-1} (sp2 aromatic C-H), 1630 cm^{-1} (sp2 amide C=O), 1570 cm^{-1} (sp2 benzene C=C), 1500 cm^{-1} (sp3 C-C)

1.3 Polymers for Comparison

This manuscript reports on 3 branched polymers containing Nile red, and a linear equivalent, which have been functionalised with vancomycin to allow biological interactions. It is designed to expand our understanding of chain architecture and polymer solvency which dictates function. To assist that we provide detailed characterisation of these polymers with previously published HB-PNIPAM materials to allow direct comparison – and also an understanding of what would be expected if the degree of branching was increased or decreased compared to what is presented, or show the properties of unfunctionalized (vancomycin free) equivalents to the data in the main manuscript.

Some additional polymers (with degrees of branching beyond that reported in this manuscript) are similar to those previously reported by Plenderleith *et al*. and Swift *et al*. in 2014 and 2016 respectively.[1,5]

Regardless of source of origin - these are highly branched PNIPAM materials that are not functionalised with vancomycin – meaning they retain acid functionality on the chain end.

Table ESI 1. Molecular Weights from Polymer Synthesis comparing this paper with samples from literature

Polymer	Label	M_n	M_W	Đ[a]	α[a]
10 : 1	None	68,469	459,510	6.71	0.08
15 : 1	None	74,263	452,073	6.09	0.15
15 : **1**	NR	260,400	769,700	2.95	0.18
25 : 1	None	103,643	588,896	5.68	0.22
25 : **1**	NR	163,900	782,700	4.77	0.16
35 : 1	None	145,131	694,943	4.79	0.22
45 : 1	None	180,729	770,721	4.26	0.24
45 : **1**	NR	183,900	537,500	2.92	0.25
55 : 1	None	178.226	706,627	3.96	0.27
65 : 1	None	143,633	560,299	3.90	0.30
75 : 1	None	354,407	958,235	2.70	0.39
85 : 1	None	298,724	908,581	3.04	0.38
Linear	None	-	-	-	-
Linear	NR	1,289,300	1,635,500	1.26	0.66

[a] M_n, M_w, Đ and α values determined by viscometric detection from GPC chromatograms.

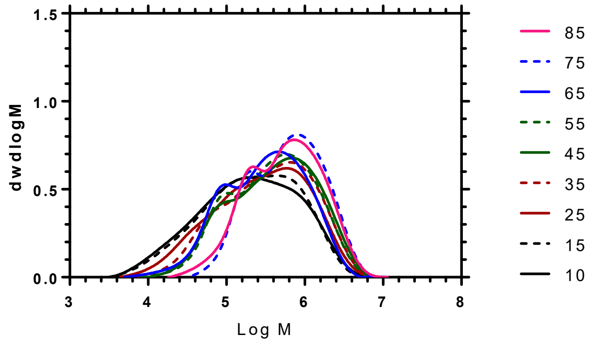

Figure ESI 1 – Molar Mass Distribution of label free polymers 10 – 85 : 1

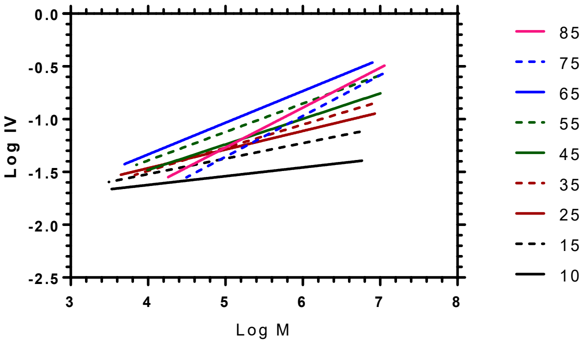

Figure ESI 2 – Extrapolated intrinsic viscosity across molar mass distribution of label free polymers 10 – 85 : **1** ratio

Table ESI 2. Fluorescence Peak Details (wavelength, λ and Intensity, I) of PNIPAM polymers in dilute solution from 10 to 45 °C

Polymer		10 °C λ / nm	Δλ / -nm	10 °C I / 10E^3 CPS	Δ I / 10E^3 CPS
15 : **1**	Pyrrole	625	2	245	-26
	Acid	622	-1	595	-91
	Vanc.	633	7	175	-7
25 : **1**	Pyrrole	640	14	458	100
	Acid	640	25	461	799
	Vanc.	641	13	177	39
45 : **1**	Pyrrole	648	18	402	209
	Acid	648	25	264	154
	Vanc.	649	14	223	90
L	Homo	658	19	136	164
	VBA	651	18	146	77
	Vanc.	648	9	121	39

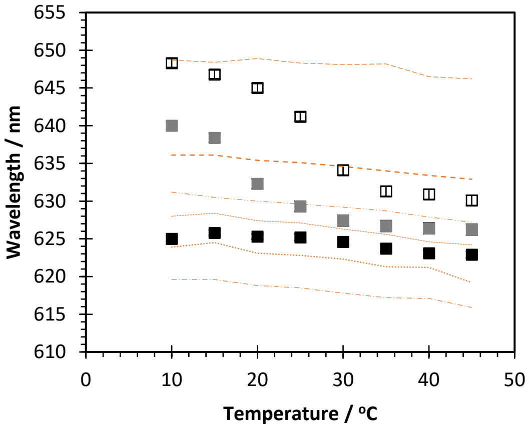

Figure ESI 3 – Shift in peak fluorescence emission wavelength (average mean of distribution) with temperature of pyrrole chain end polymers compared to ethylene glycol, methanol, ethanol, butanol, isopropanol and DMF solvent shifts. Branching ratio 15 : 1 black, 25 : 1 grey and 45 : 1 clear.

Table ESI 3. Cloud point of highly branched polymers (°C)

Ratio of NIPAM : RAFT	Linear	45 : **1**	25 : **1**	15 : **1**
Unmodified	35.5	27.9	22.5	20.0
Acid derivitised	33.0	29.0	26.0	29.5
Vanc. derivitised	35.0	-	-	-
*Above in PBS	15.3	17.1	16.3	16.5
*Above with Ala-Ala	13.6	14.8	14.9	14.7

Table ESI 4. LCST ($T_{c\text{-}g}$ °C) of highly branched polymers (°C)

Ratio of NIPAM : RAFT	Linear	45 : **1**	25 : **1**	15 : **1**
Unmodified	33.0	24.4	19.2	12.0
Acid derivitised	34.0	27.1	22.2	15.4
Vanc. derivitised	36.0	33.9	32.0	27.0
*Above in PBS	33.9	29.9	29.0	21.5
*Above with Ala-Ala	34.1	30.2	29.3	19.8
Δq of peptide / mJ [a]	1.4	4.6	5.4	0.2

[a] – Data on bottom row is reduction of enthalpy of LCST caused by presence of Ala-Ala peptide.

1.4 Nuclear Magnetic Resonance Analysis

Raw data for ^{1}H NMR listed in polymer synthesis sections are shown below in **ESI1**.

1

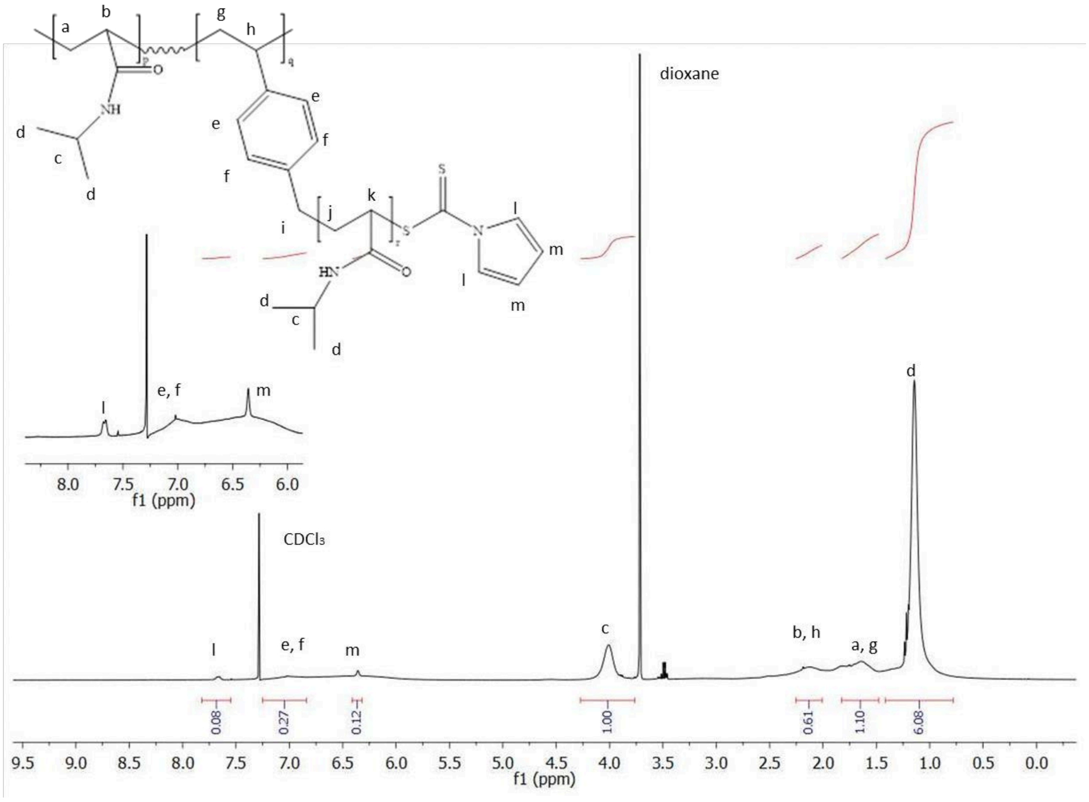

2

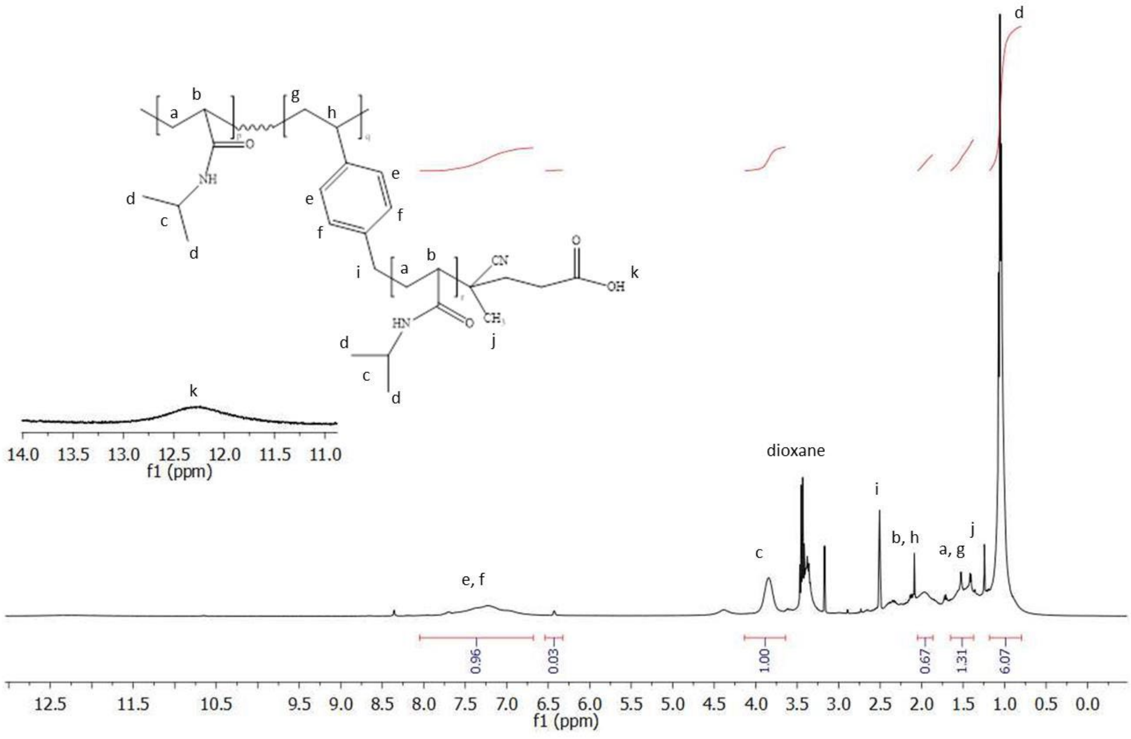

3

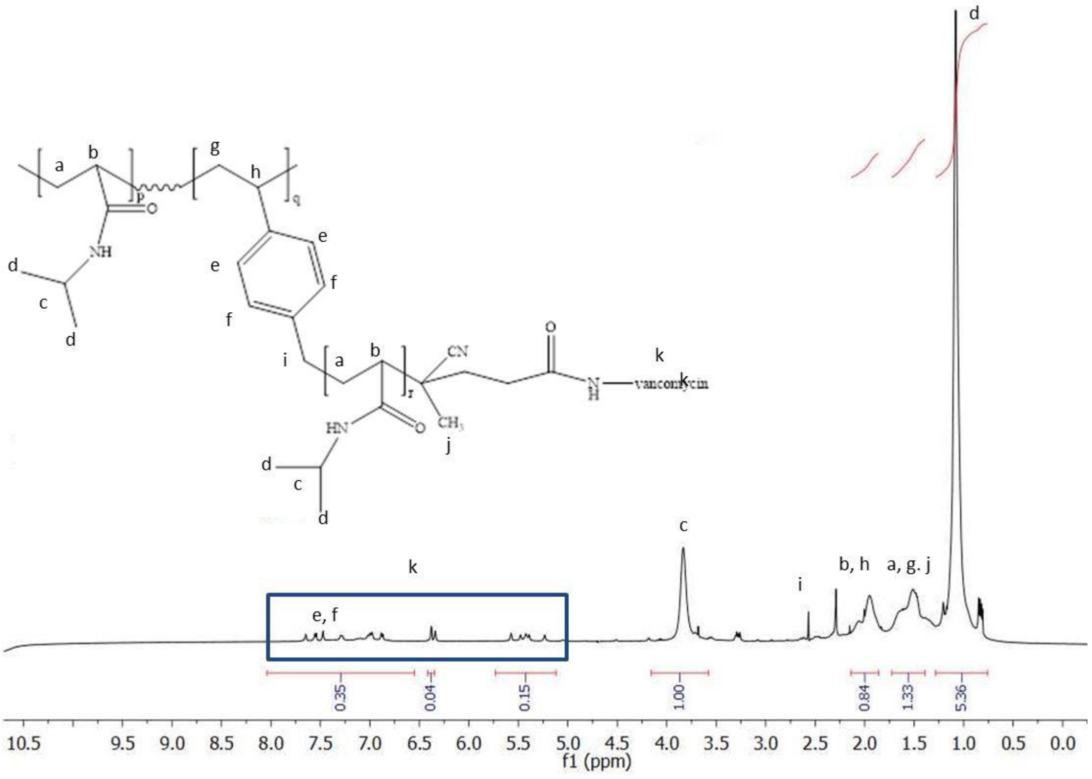
d
a
b
g
h
NH
e
f
c
i
k
CN
vancomycin
HN
CH3
j
e, f
b, h
a, g. j
0.35
0.04
0.15
1.00
0.84
1.33
5.36
10.5
9.5
9.0
8.5
8.0
7.5
7.0
6.5
6.0
5.5
5.0
4.5
4.0
3.5
3.0
2.5
2.0
1.5
1.0
0.5
0.0
f1 (ppm)

4

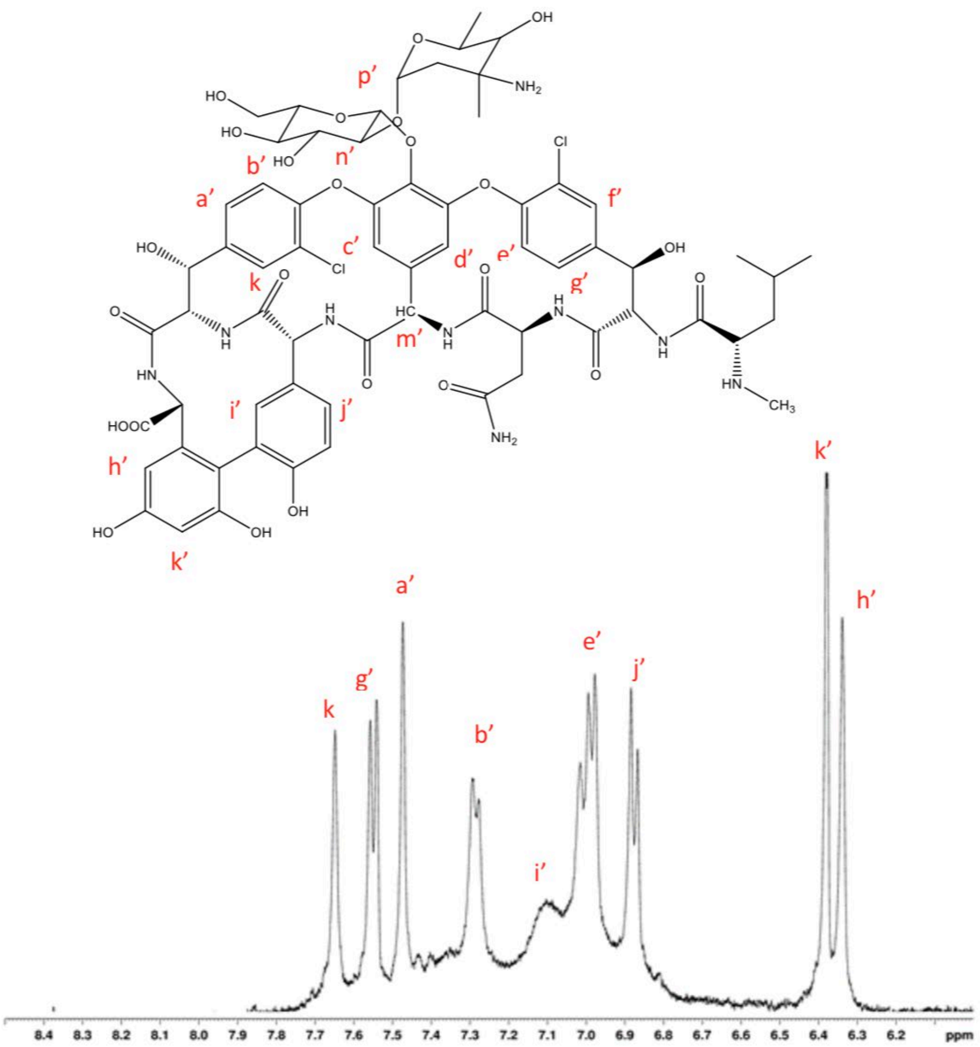
p’
n’
b’
a’
c’
d’
e’
f’
g’
k
m’
i’
j’
h’
k’
HOOC
NH2
Cl
OH
HN
CH3
k’
h’
a’
e’
j’
g’
k
b’
i’
8.4 8.3 8.2 8.1 8.0 7.9 7.8 7.7 7.6 7.5 7.4 7.3 7.2 7.1 7.0 6.9 6.8 6.7 6.6 6.5 6.4 6.3 6.2
ppm

5

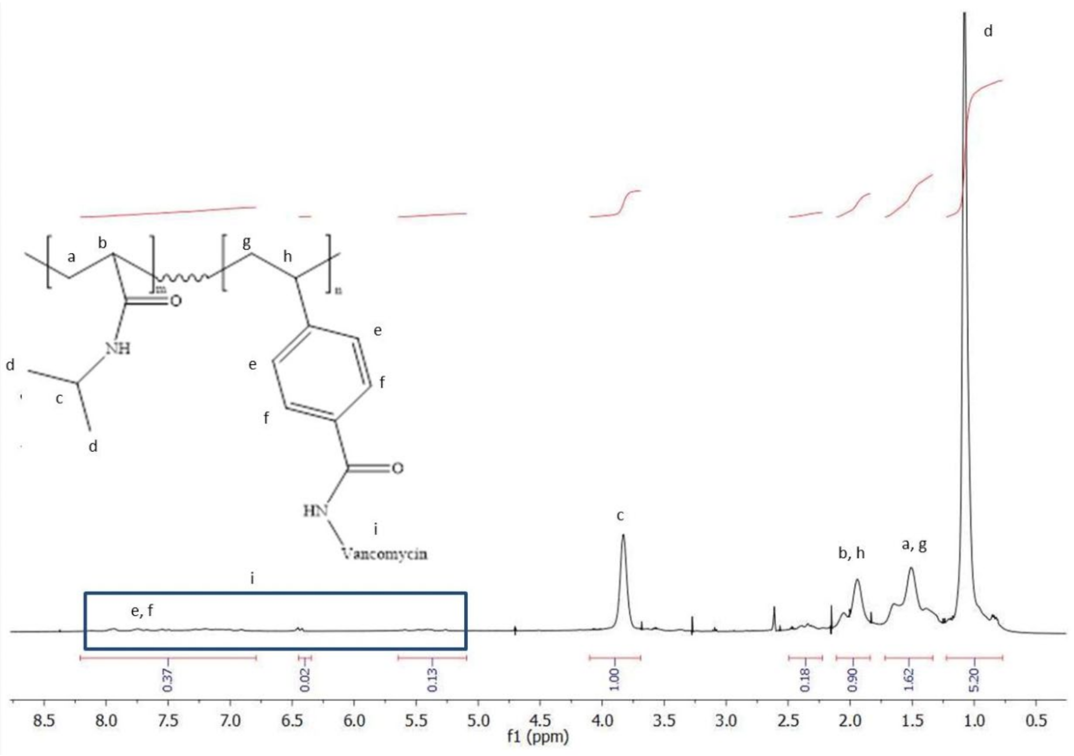

6

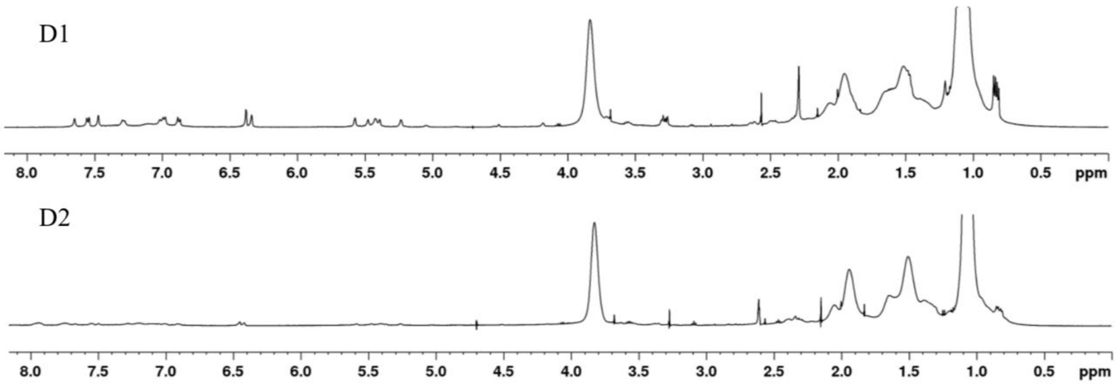

Figure ESI 4 – **1**) ^{1}H NMR spectrum of **2^a** (HB-PNIPAM with pyrrole end groups) with the expanded region showing the peaks due to the pyrrole groups (m and l) and the peaks due to the styryl groups (e and f) **2**) ^{1}H NMR spectrum of 2^b (HB-PNIPAM-COOH), with the expanded region showing the peaks due to the carboxylic acid groups (k) **3**) ^{1}H NMR spectrum of **2** (HB-P(NIPAM-co-NR)-van) in D_2O **4**) Expansion of ^{1H} NMR spectrum of 2 ((δ=6.4-8.0)) to show vancomycin drug functional signals **5**) ^{1}H NMR spectrum of **4** (L-PNIPAM-van) in D_2O **6**) Expanded ^{1}H NMR of (D1) **2** and (D2) **4** for easy visual comparison.

NMR analysis of these precursor polymers allows for determination of branching chain end fidelity to be determined. The following section uses methodology discussed with the ESI of Plenderleith *et al to determine the chain end functionality of the precursor polymers*.[1] [H] functionality calculations using integrals of the NIPAM isopropyl functional unit, the benzyl unit and the N-pyrrole hydrogens were compared, fixing the isopropyl NIPAM functionalities to 1. The ^{1}H values were calculated by dividing the integrals of the isopropyl NIPAM by 6, Benzyl by 4 and N-pyrrole by 2. Distal hydrogen from the pyrrole functional unit were removed from the overlapping benzyl integrals prior to calculation. Calculations for functionality were used to calculate the % pyrrole chain ends per branch point and the degree of branching (number of branch points per number of monomers).

$$\%\ Pyrrole\ Functionality = \frac{[H]_{Pyrrole}}{[H]_{Benzyl}} \times 100$$

To determine the ratio of acid chain ends TAI (trichloroacetyl isocyanate) was added to the polymers within the NMR tube. This produced an imide peak at 11.1 ppm that could be quenched if D_2O was added.

$$\%\ Acid\ Functionality = \frac{[H]_{Imide}}{[H]_{Benzyl}} \times 100$$

Table ESI 5. Chain end fidelity of precursor polymers determined via NMR analysis.

**Polymer**	**Conv (%)**	**Pyrrole (%)**	**Acid (%)**	**DB**
**1**[a]	98	47	11	0.06
**2**[a]	98	53	14	0.035
**3**[a]	98	51	27	0.023

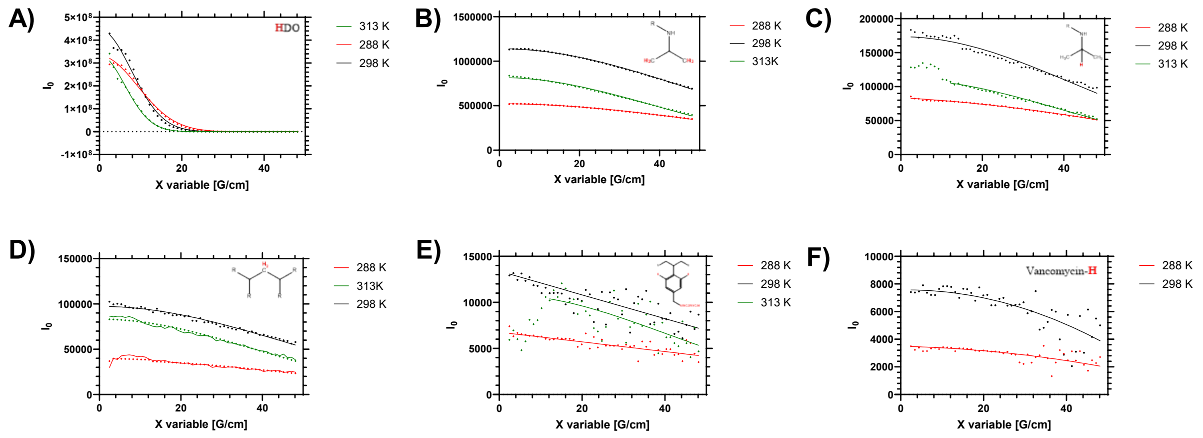

Figure ESI 5 – Stejskal-Tanner plots of high resolution DOSY (48 gradient decay steps) of Linear-PNIPAM-Vanc (Polymer **4)** in D_2O at 288, 298 and 313 K. Data is direct comparison to Figure 2 in manuscript that represents polymer **2** (25 : 1 branching). These represent peaks at A) 4.69 ppm (H_2O residue in solvent), B) 1.1 ppm (-C$\mathbf{H_3}$ in PNIPAM isopropyl side chain), C) 3.8 ppm (-C**H**-$(CH_3)_2$ in PNIPAM isopropyl side chain), D) 1.5 ppm (CH_2 in polymer backbone), E) 7.4 ppm (Aromatic peaks from benzyl branching group), F) 6.47 ppm (Vancomycin aromatic-H peak from functional chain ends). Data at 313 K subject to poor shimming (due to sample turbidity) that affects quality of lower intensity peaks at low G/cm so only higher magnetic gradient portions of spectra are used in fitting processed.

Table ESI 6 – Intensity and Diffusion of ^{1}H signals from Linear-PNIPAM -Vanc in D_2O

	Peak Intensity (Integrated to solvent (4.7 ppm) peak)					
Temp (K)	1.1 ppm	1.5 ppm	3.8 ppm	4.7 ppm	6.5 ppm	7.4 ppm
**288**	1.07	0.33	0.24	100	0.03	0.03
**298**	1.03	0.32	0.23	100	0.03	0.03
**313**	0.98	0.31	0.21	100	<0.01*	0.01
**Δ** [a]	8%	6%	12.5%	-	>90*	66%

	Peak Diffusion (x 10^{-10} M^2 S^{-1})					
Temp (K)	1.1 ppm	1.5 ppm	3.8 ppm	4.7 ppm	6.5 ppm	7.4 ppm
**288**	0.34	0.34	0.35	10.92	0.34	0.32
**298**	0.44	0.46	0.49	15.6	0.44	0.47
**313**	0.60	0.63	0.60	24.0	*	0.59

* Data for the vancomycin peak at 6.5 ppm shifts from 6.4 to 6.8 ppm as the polymer collapses. It is very small and no accurate diffusion value can be determined due to the poor quality of the fit.

[a] Δ is the % reduction of integrated proton signal for broad ^{1}H peak as sample is heated from 288 to 313 K. This is distinct from initial intensity of proton signal in Stejskal-Tanner plots (Figure 2) which only shows proton intensity at exactly peak centre (precise ppm matching) and does not account for peak broadening.

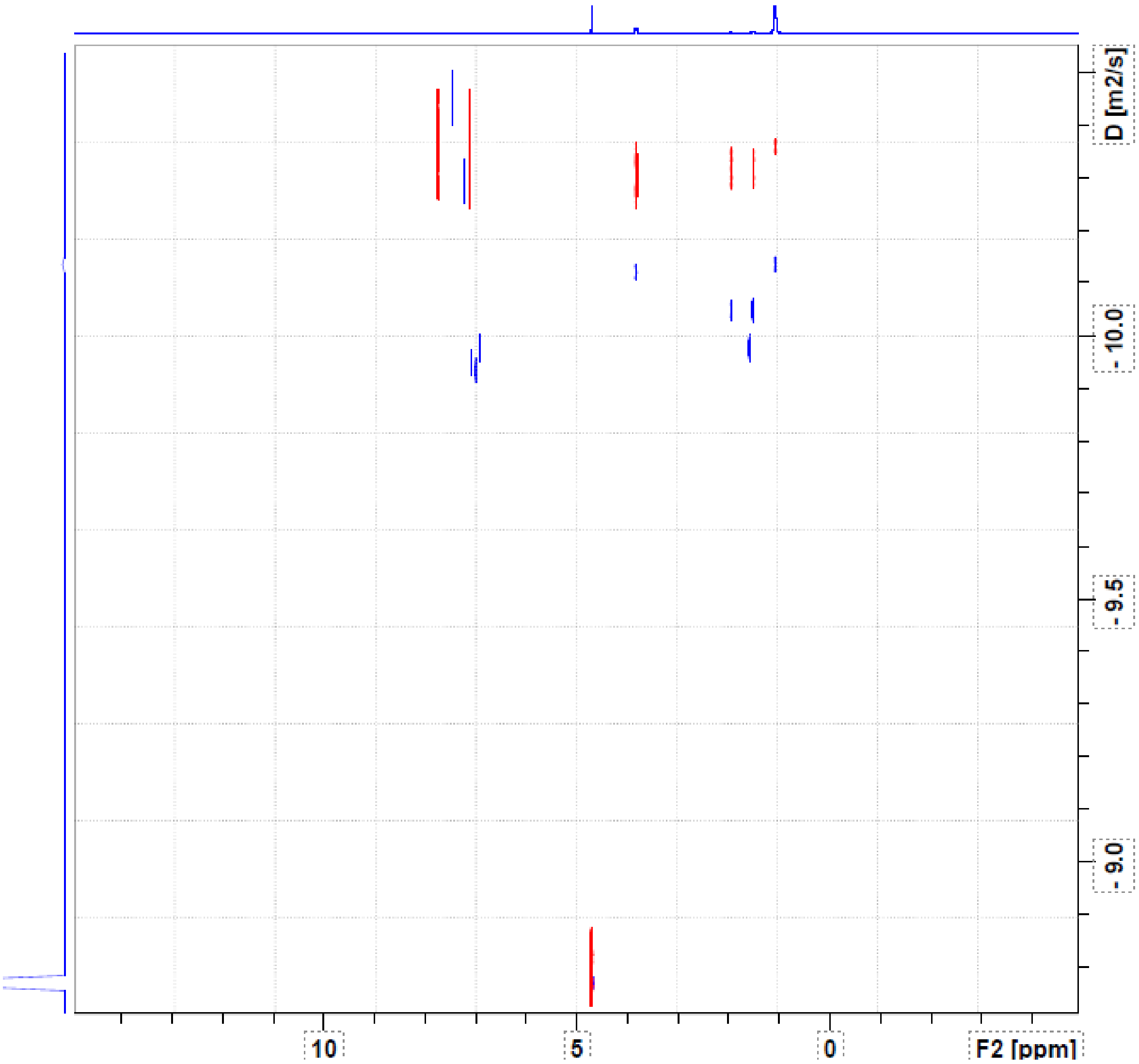

Figure ESI 6 – Comparison of processed DOSY spectra of Polymer **2** (branched polymer - blue) and **4** (linear polymer - red) in D2O at 298 K. This extrapolates data from **Figure 2** (Polymer **2**) and **Figure ESI 5** (Polymer **4**) – showing the overlap of diffusion peaks for the linear polymer and the dispersity of diffusion peaks observed in the branched polymer system.

1.5 FTIR Analysis

Raw data used to provide FTIR peaks listed in the chemistry synthesis section are shown in **ESI 7**.

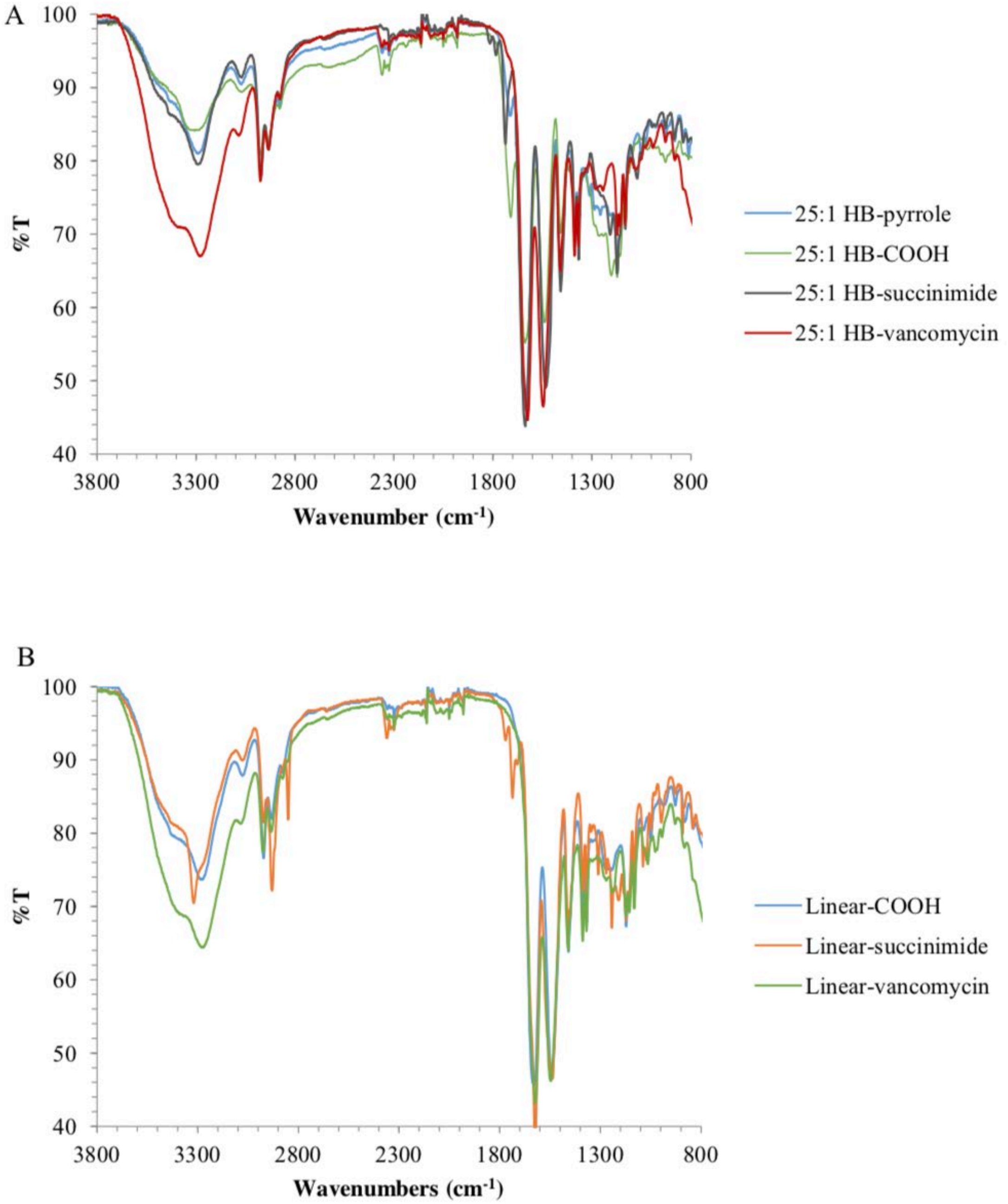

Figure ESI 7 – **A**) FTIR raw data of polymers 2^a, 2^b, a 2 succinimide precursor and **2**. **B**) FTIR raw data of polymers 4^b, a 4 succinimide precursor and **4**.

1.6 Polymer Molar Mass Analysis and Vancomycin Loading

The molar masses of polymers 1-4 were measured using Universal SEC, using 2x polargel columns, using pre-filtered methanol as a mobile phase. The isocratic pump pressure was approximately 4000 kPa, with a ripple of 0.51, and the viscometer internal pressure (IP) was set at 45 kPa. The column set had a hold up volume of 33.2 cm^3 and an internal pore volume of 19.74 cm^3 determined from the retention time of a non-retained molecule, toluene, and fully excluded high molar mass PNIPAM.[5] Samples were prepared in pre-filtered Methanol (1 mg ml^{-1}) and injected through stationary phase columns (flow rate 1 ml min^{-1}) maintained at 30 ◦C. This was then fed through Agilent refractive index and viscometric (Agilent 1260 Infinity Detector Suite) detectors each maintained at 30 ◦C. The system was calibrated using PNIPAM standards of known molar mass.[5] The raw data of this analysis, the calibration standards of this system, and the determined molar mass distributions of the polymers are shown in **Fig ESI 8**. These are large molar mass materials and as they do not represent unimodal normal distributions it is not entirely reasonable to describe them purely using molar mass moments. As such we have also described the material using the non-parametric centiles, taken from the cumulative distribution and presented this data as well.

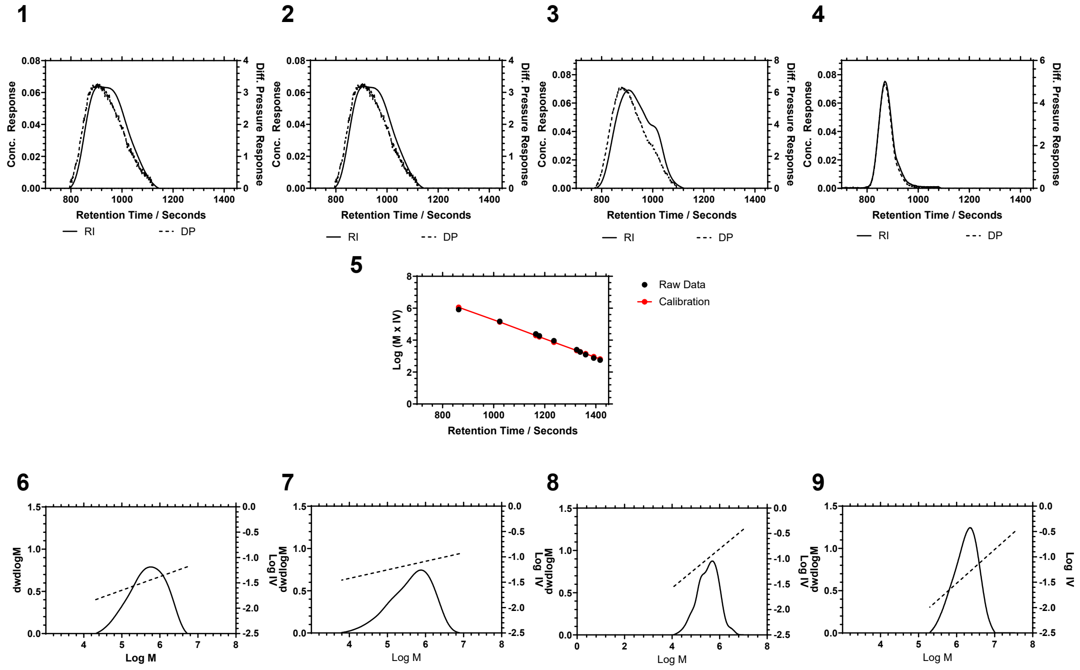

Figure ESI 8 - MeOH GPC Universal SEC analysis of polymers, system calibrated with linear PNIPAM standards[26]. **1-4**) show raw RI and DP responses of polymers **1-4**, **5**) shows response of linear PNIPAM standards used to calibrate SEC system, **6-10**) Molar mass distribution (solid line) and intrinsic viscosity (dashed line) of nile red containing vancomycin functionalised polymers **1**, **2**, **3 4.**

Table ESI 7. Characterisation of Polymers **1-3** molar mass distribution via interquartile ranges

	1st quartile (kg mol^{-1})	Median (kg mol^{-1})	3rd quartile (kg mol^{-1})
15 : **1**	348	642	1,077
25 : **1**	179	498	1,098
45 : 1	163	353	700

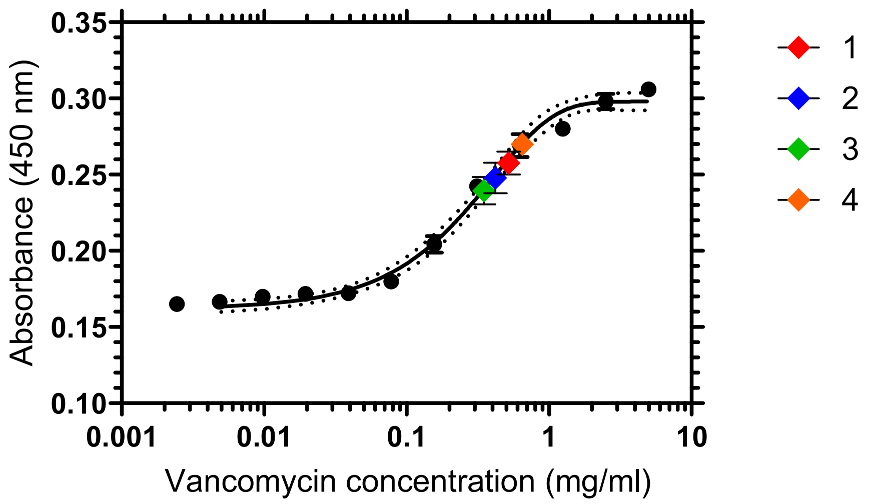

Figure ESI 9. Standard curve of vancomycin and quantification of the amount of vancomycin in polymers. The curve was fitted by sigmoidal, 4-parameter logistic fit (4PL) of the data points. X is log(concentration) to determine drug loading.

1.7 Polymer Cloud point + LCST Studies

As the polymers show a lower critical solution temperature this was studied both via turbidimetry (cloud point) and micro-DSC (LCST).

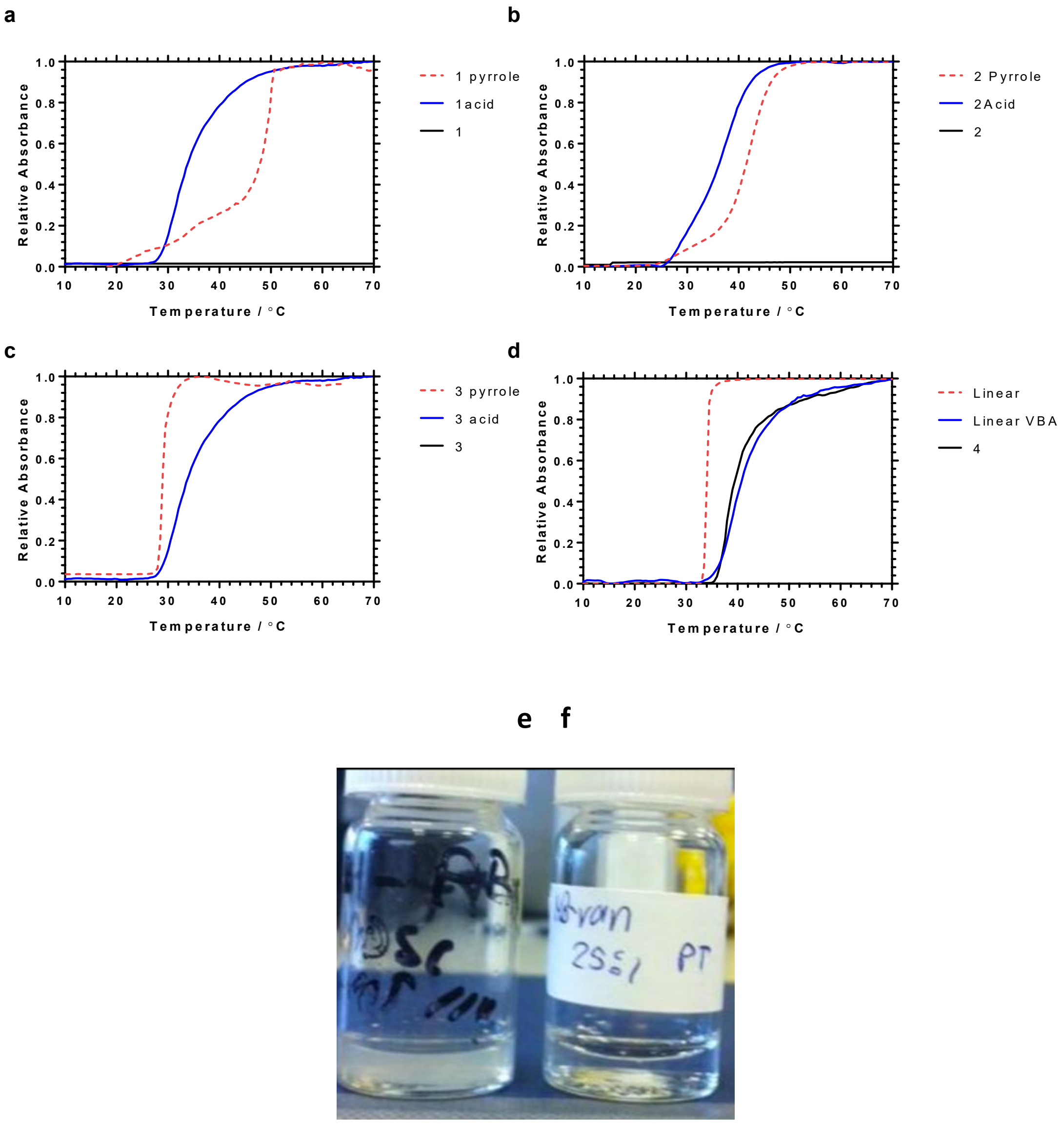

Figure ESI 10 – **a – d)** Turbidometry response of 1 mg ml^{-1} PNIPAM in solution across LCST in ultrapure water **e –f)** Visual comparison of 10 mg ml^{-1} **4** (left) and **2** (right) in deionised water at 37 °C

Table ESI 8 - Cloud point (turbidity) and LCST (calorimetry) results summary of highly branched polymers, and the effect of ionisable groups from vancomycin on the colloidal stability of HB-PNIPAM-van in solution above the LCST.

	Cloud Point				Lower Critical Solution Temp			
	**1**	**2**	**3**	**4**	**1**	**2**	**3**	**4**
**1-4**[a] [$]	20.0	22.5	27.9	35.5	12.0	19.2	24.4	33.0
**1-4**[b] [$]	29.5	26.0	29.0	33.0	15.4	22.2	27.1	34.0
**1-4** [$]	- -	- -	- -	35.0	27.0	32.0	33.9	36.0
**1-4** *	16.5	16.3	17.1	15.3	21.5	29.0	29.9	33.9
**1-4** + Ala-Ala*	14.7	14.9	14.8	13.6	19.8	29.3	30.2	34.1
**Δq 1-4** + Ala-Ala*[a]					0.2	5.4	4.6	1.4

[$] carried out in ultrapure water (18.2 MΩ-cm) * Cloud points of polymer in PBS solution [a] Data on bottom row is reduction of enthalpy of LCST caused by presence of Ala-Ala peptide.

pH of solution	pKa = 2.2	pKa = 8.8	pKa = 10-12	Opacity (at 40°C)
pH =2	-COOH	—$\overset{+}{N}H_2$—CH_3	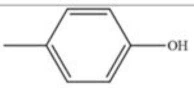	Cloudy
pH = 7.6	$-COO^-$	—$\overset{+}{N}H_2$—CH_3	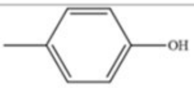	Clear

Table ESI 8 contains data for unmodified, acid derivitised, vanc. derivitised materials in aqueous solution, alongside a vanc. derivitised desolvation in PBS alone and with a low concentration of D-Ala-D-Ala peptide. The branched vancomycin functionalised polymers exhibit no cloud point in pure water due to competing electrostatic interactions[7], however still undergo an internal LCST rearrangement on heating, and full aggregation can be restored by switching the solvent to PBS. Micro DSC data indicates the binding of Ala-Ala peptides to chain ends does not alter the LCST temperature of the polymer – although they have a significant effect on the enthalpic energy released and do affect the aggregation potential of the desolvating polymer (hence why reduction in cloud points are observed in Table 3). Polymers **2** and **3** polymer demonstrate significant energy release compared to the linear polymer due to an increased number of binding sites. The change in enthalpy for **1** is much smaller however this still represents a large % of the total enthalpy released by this polymer which is already largely desolvated across the temperature range.

1.8 Thermal Degradation of Polymer Chain Ends

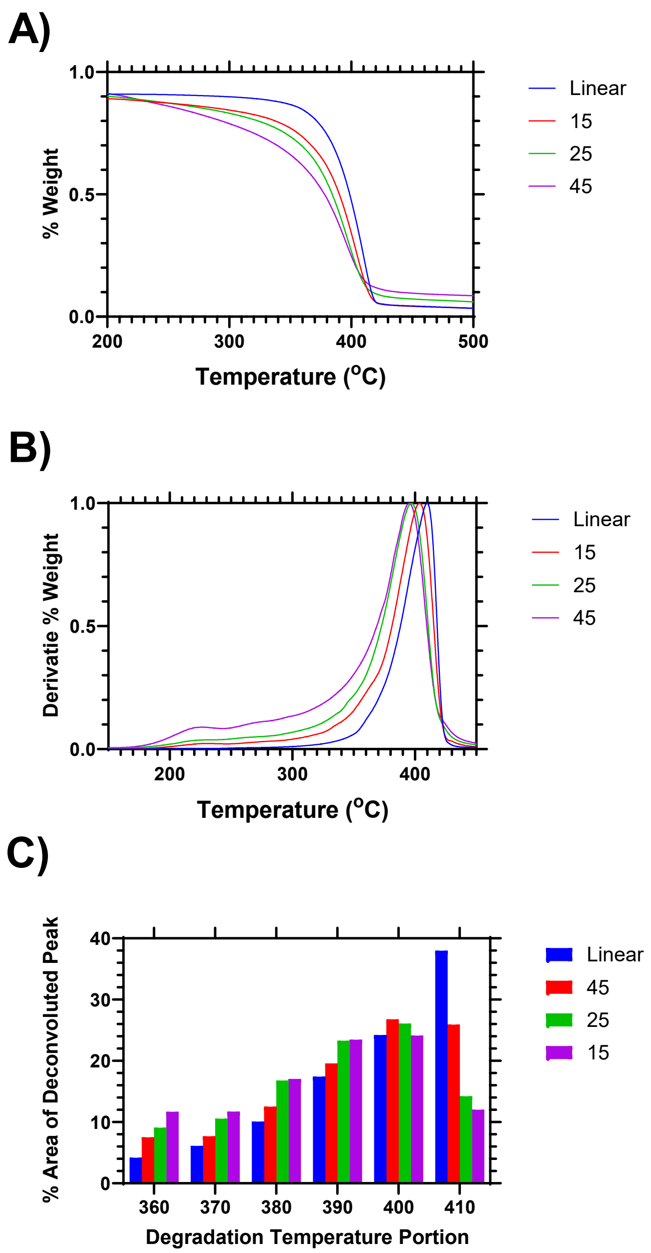

Figure ESI 11. A) Thermogravimetric Analysis (TGA) mass loss profiles for precursor (unmodified) polymers synthesised with varying degrees of branching. B) Derivitive of mass loss with temperatures. C) %TTGA (area deconvoluted peak) for 10 °C temperature ranges across second observed degradation step (360 – 410 °C).

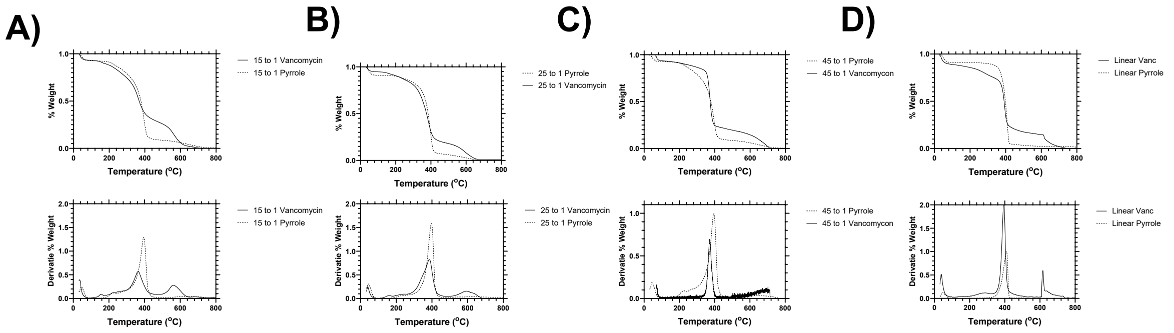

Figure ESI 12. A) TGA of polymer **1** and $\mathbf{1}_{\text{precursor}}$ (with residual pyrrole chain ends) B) TGA of polymer **2** and $\mathbf{2}_{\text{precursor}}$ (with residual pyrrole chain ends) C) TGA of polymer **3** and $\mathbf{3}_{\text{precursor}}$ (with residual pyrrole chain ends) D) TGA of polymer **4** and $\mathbf{4}_{\text{precursor}}$ (with residual pyrrole chain ends)

1.9 Additional Diffusion NMR Analysis

1.8.1 Aqueous solvents:

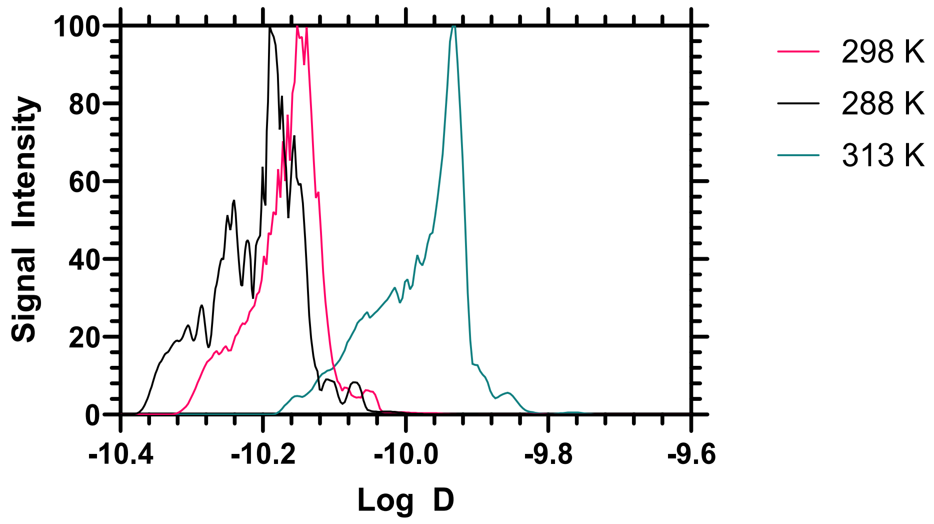

Figure ESI 13 – Diffusion distribution (precursor raw data for Figure 3 in main manuscript)

1.8.2 Organic Solvents:

A)

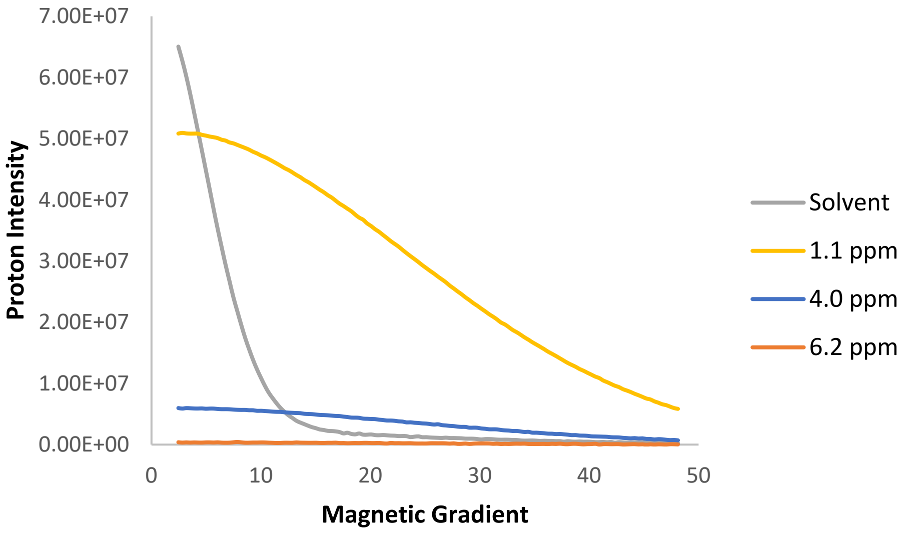

B)

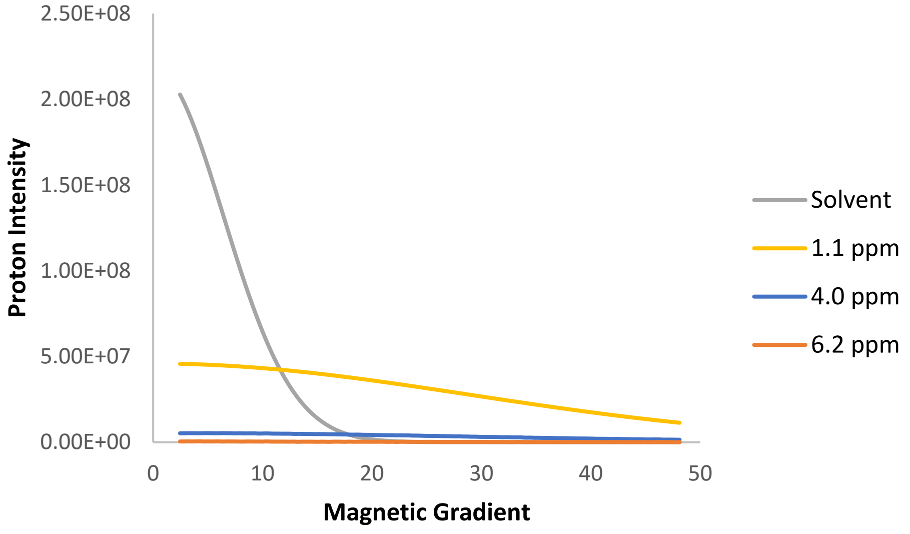

2.50E+08
2.00E+08
1.50E+08
1.00E+08
5.00E+07
0.00E+00
Proton Intensity
0
10
20
30
40
50
Magnetic Gradient
Solvent
1.1 ppm
4.0 ppm
6.2 ppm

C)

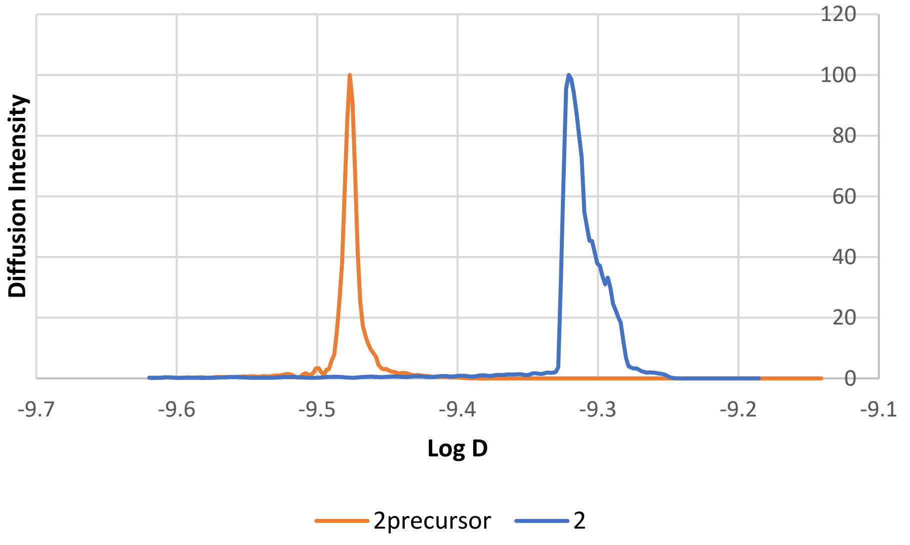

Diffusion Intensity
120
100
80
60
40
20
0
-9.7
-9.6
-9.5
-9.4
-9.3
-9.2
-9.1
Log D
2precursor
2

D)

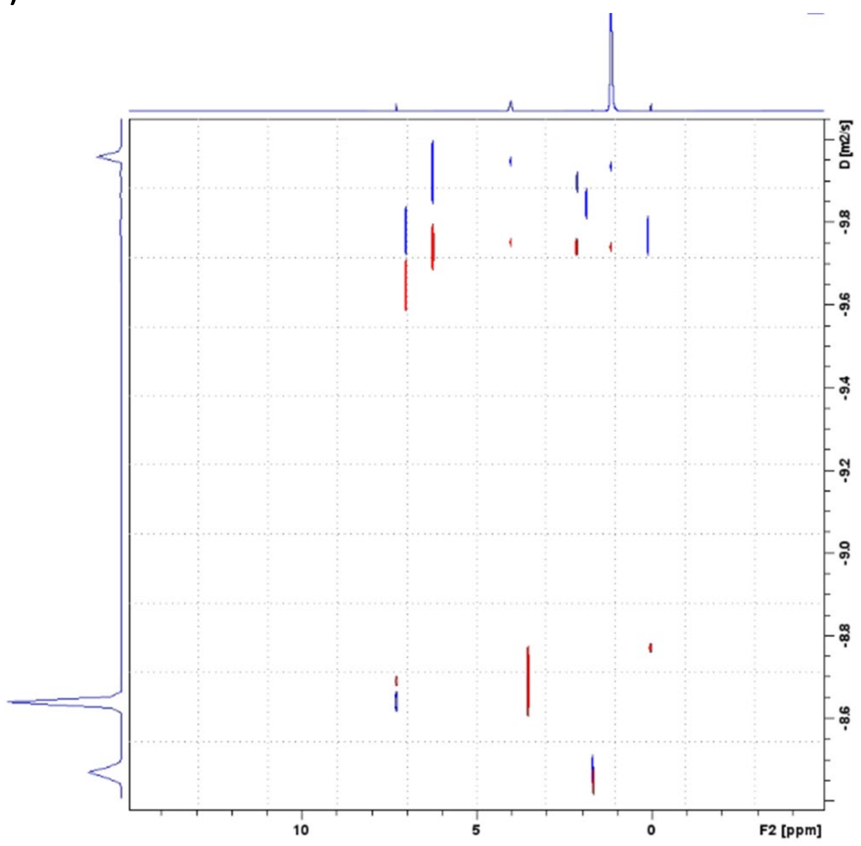

Figure ESI 14 – Analysis of NMR data for Polymer **2** and $\mathbf{2}_{precursor}$ in $CDCL_3$.
A-B) Stejskal-Tanner plots of polymer $\mathbf{2}_{precursor}$ (A) and **2** (B) in $CDCL_3$**. C)** Projected sum of Diffusion distribution of $\mathbf{2}_{precursor}$ and **2**. **D)** DOSY plot of $\mathbf{2}_{precursor}$ and **2**.
Peak **D** values of $\mathbf{2}_{precursor}$: 1.16 ppm: 1.71 x 10^{-10} M^2 S^{-1}, 4.02 ppm: 1.67 x 10^{-10} M^2 S^{-1}, 6.23 ppm: 1.73 x 10^{-10} M^2 S^{-1}. Solvent: 3.49 x 10^{-9} M^2 S^{-1}
Peak D values of **2**: 1.16 ppm: 1.11 x 10^{-10} M^2 S^{-1}, 4.02 ppm: 1.07 x 10^{-10} M^2 S^{-1}, 7.01 ppm: 1.58 x 10^{-10} M^2 S^{-1}. Solvent: 2.24 x 10^{-9} M^2 S^{-1}

1.10 Ab Initio Modelling

DFT Modelling of Nile Red Analogues

Several variations of Nile red were modelled to predict the electron distribution in the frontier molecular orbitals. These calculations were carried out using the ab initio chemistry package ORCA,[8] which has been shown to be a consistent and efficient computational system for studying DFT (Density Functional Theory) and TD-DFT (Time-Dependent DFT) ab initio simulations of absorption / emission processes of organic molecules.[9]

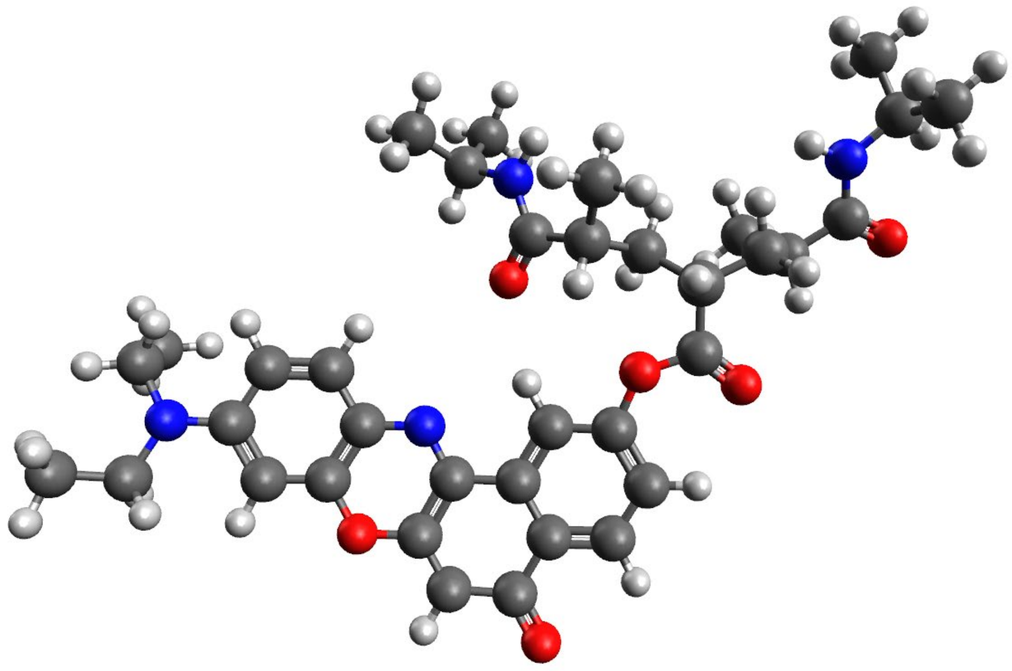

Figure ESI 15 – Structure of Nile Red-*co*-NIPAM oligomeric segment used to modelling calculations.

Table ESI 9 – eV predicts of transitions to S_1, S_2 and S_3 excited states of structures in gas state and with different CPCM environments.

Nile Red	CPCM Water	CPCM Methanol	CPCM Ethanol	CPCM Octanol	CPCM Hexane	Gas State
S_1	2.568	2.573	2.564	2.559	2.656	2.885
S_2	3.141	3.144	3.144	3.149	3.017	2.892
S_3	3.246	3.236	3.230	3.201	3.199	3.254
**Nile Red Acrylate**	**CPCM Water**	**CPCM Methanol**	**CPCM Ethanol**	**CPCM Octanol**	**CPCM Hexane**	**Gas State**
S_1	2.543	2.548	2.540	2.535	2.630	2.847
S_2	3.06	3.062	3.062	3.066	3.006	2.867
S_3	3.148	3.152	3.154	3.165	3.106	3.150
**Nile Red Monomer**	**CPCM Water**	**CPCM Methanol**	**CPCM Ethanol**	**CPCM Octanol**	**CPCM Hexane**	**Gas State**
S_1	2.563	2.567	2.557	2.549	2.633	2.852
S_2	3.108	3.110	3.110	3.112	3.023	2.884
S_3	3.259	3.247	3.241	3.21	3.199	3.179
**Nile Red *co* NIPAM**	**CPCM Water**	**CPCM Methanol**	**CPCM Ethanol**	**CPCM Octanol**	**CPCM Hexane**	**Gas State**
S_1	2.569		2.565		2.659	
S_2	3.128		3.132		3.051	
S_3	3.270		3.254		3.194	

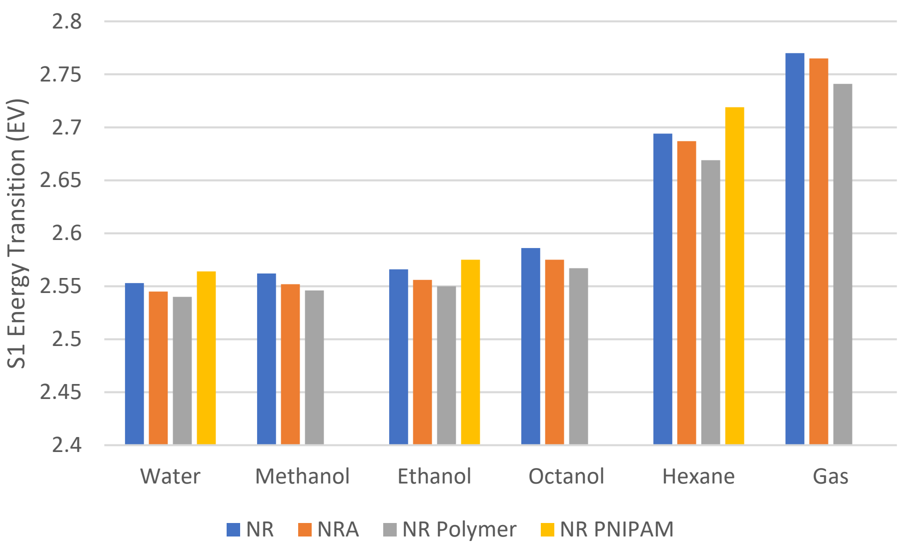

A)

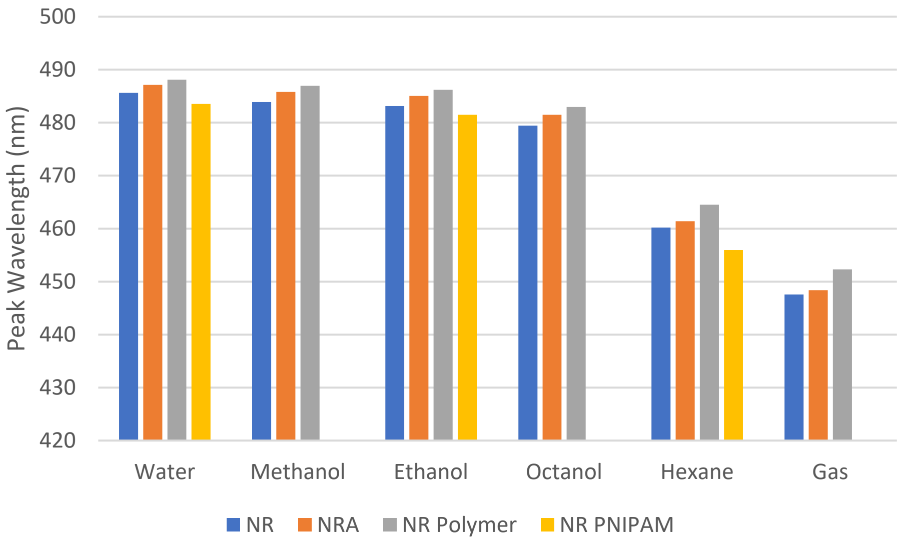

B)

Figure ESI 16 - HOMO-LUMO Band Gap (S1 transition) of Nile Red, Nile Red Acrylate, Nile Red Monomer and Nile Red co- NIPAM oligomer with different CPCM force fields applied. A) shows difference in electron volts, B) suggests indicative absorption wavelength in nm.

1.11 Fluorescence of PNIPAM NR

Nile red acrylate shows a strong colourometric shift when exposed to different solvents: it is a pale yellow in diethyl ether, bright pink in acetone and a blue/purple in a mixture of methanol and water (**ESI17a**). This is reflected by the shift in the emission wavelength following excitation. The determined fluorescence emission wavelength in **Figure 6** come from a broad emission spectrum fitted to a Gaussian distribution (**ESI17b**). The fluorescence output of precursor polymers (1-4a (*initial polymer precursor*) and 1-4b (*polymer intermediates modified with an increased concentration of acid chain end by cleaving imidazole RAFT groups*) were analysed in addition to **1-4** (as shown in **Figure 1c + d**) which are shown in **ESI17e-j**. These show that the fluorescence response of the NR (covalently bonded within the polymer coil) is highly dependent on the chain ends of the branched polymer material, as that in turns alters the conformational properties and desolvation state of the polymer chains[7]. The fluorescence lifetime of these materials however is independent of wavelength of emission > 620 nm (**ESI17d**) and so the same fluorescence emission wavelength can be used to compare all four polymers in **Fig 6**.

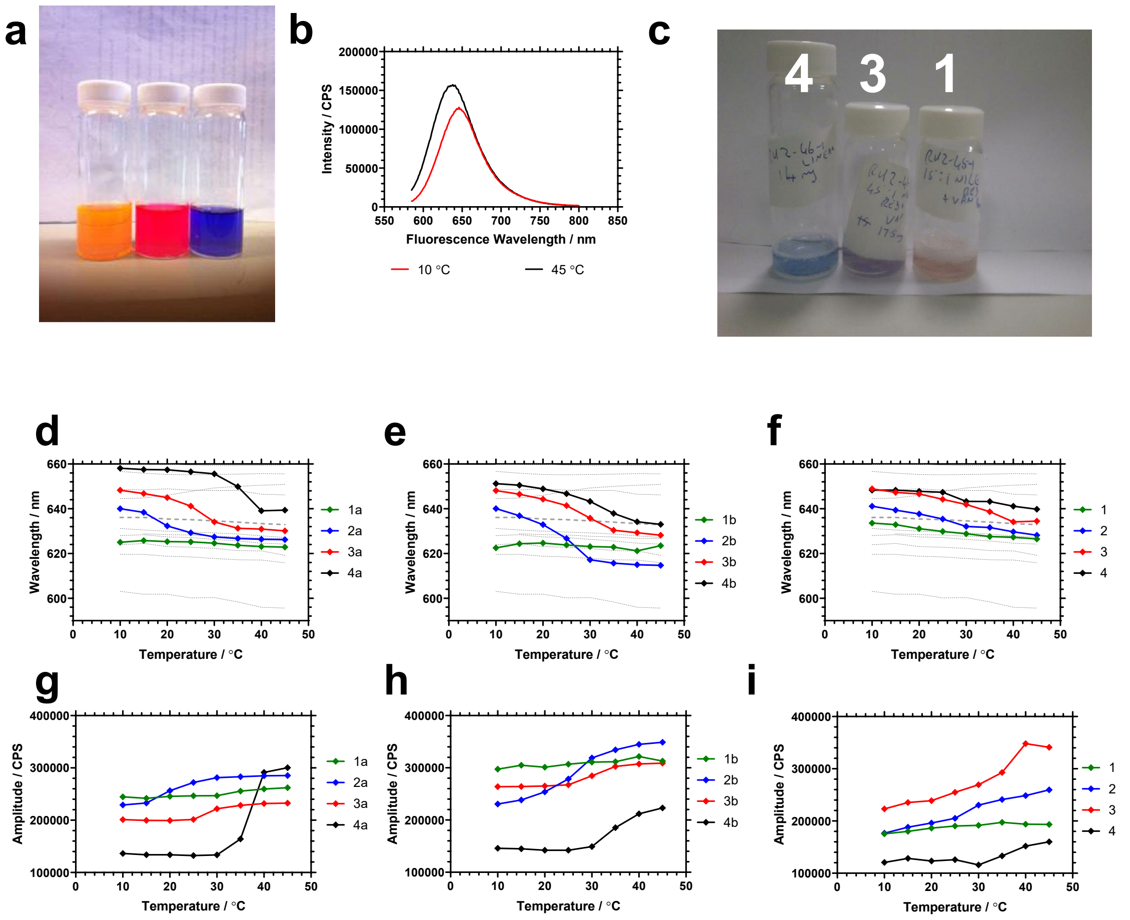

Figure ESI 17 – **a)** Photograph of NR (0.001 mg ml^{-1}) in diethyl ether, acetone and methanol/water mixture). **b)** Emission of **4** (1 mg ml^{-1}) in water following excitation at 580 nm carried out at 10 °C (red) and 45 °C (black). **c)** Photograph of **1**, **3** & **4** solutions (1 mg ml^{-1}) in water at 28 °C. **d-f**) Shift in peak fluorescence emission wavelength (average mean of distribution) with temperature of dilute 1 mg ml^{-1} solutions of d) **1-4^{a}**, e) **1-4^{b}** and f) **1-4**, compared to ethylene glycol, methanol, ethanol,

butanol, isopropanol and DMF solvent shifts. Colours: **1** green, **2** blue, **3** red, **4** black. **g-i)** Increase in amplitude of peak fluorescence emission (average of distribution) with temperature of h) **1-4**[a], i) **1-4**[b] and j) **1-4**. Colors: **1** green, **2** blue, **3** red, **4** black.

The fluorescence experiments outlined in **Fig 6** and **ESI 7** were carried out in water, whilst most biological systems do not exist in a salt free solution. As such additional fluorescence experiments were carried out on the polymers in PBS (**ESI18a-b**) to observe the different fluorescence response of the system. It was found that in PBS the more linear polymers in PBS the nile red dye of the polymers exhibited a slightly less polar environment below the LCST than was observed for the same solutions in water (**ESI18c**), and the more highly branched polymers were not as greatly affected by the presence of salt. In conclusion in the presence of salt polymers **1-4** exhibited broader thermochromic type-shifts over a broader temperature range than seen in the ultrapure aqueous solution.

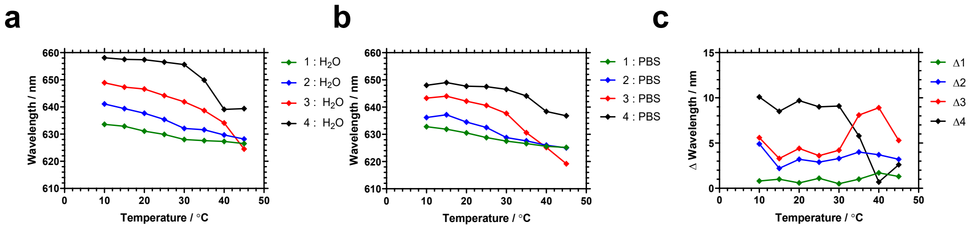

Figure ESI 18- a-c) Shift in peak fluorescence emission wavelength (average mean of distribution) of polymers **1 – 4** following λ_{ex} 580 nm. Branching ratio differs by color: **1** (green), **2** (blue), **3** (red) and **4** (black). Fig a) in pure water, b) in PBS and c) shows difference between the two aqueous solutions.

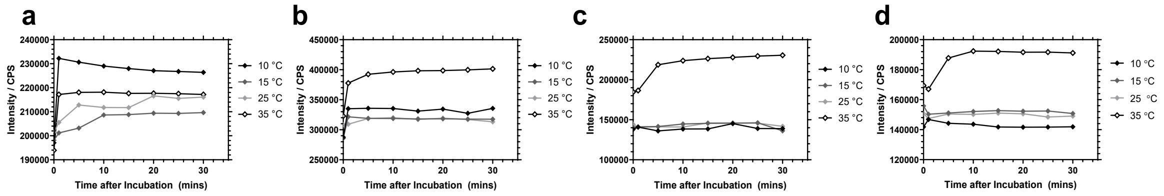

e

Polymer	Δ	10 °C	15 °C	25 °C	35 °C
**1**	**ΔI** / ΔT	**35** / 0	**10** / 15	**15** / 20	**25** / 5
**2**	**ΔI** / ΔT	**50** / 5	**35** / 0	**10** / 5	**80** / 5
**3**	**ΔI** / ΔT	**5** / 20	**5** / 15	**0** / -	**45** / 5
**4**	**ΔI** / ΔT	**5** / 0	**0** / -	**0** / -	**23** / 5

Figure ESI 19 – **a–d)** Shift in peak fluorescence intensity with temperature of vancomycin chain end polymers progressing post Ala-Ala addition. Plots: a) **1** b) **2** c) **3** d) **4** in PBS (5 mg ml^{-1}) incubated with peptide (1 mg ml^{-1}). Temperatures 10 °C (black), 15 °C (dark grey), 25 °C (light grey) and 35 °C (clear). **e)** Increase in fluorescence peak intensity (I / 10E3 CPS) and time taken to reach peak intensity (T / minutes) of PNIPAM polymers on Ala-Ala Peptide binding at different temperatures.

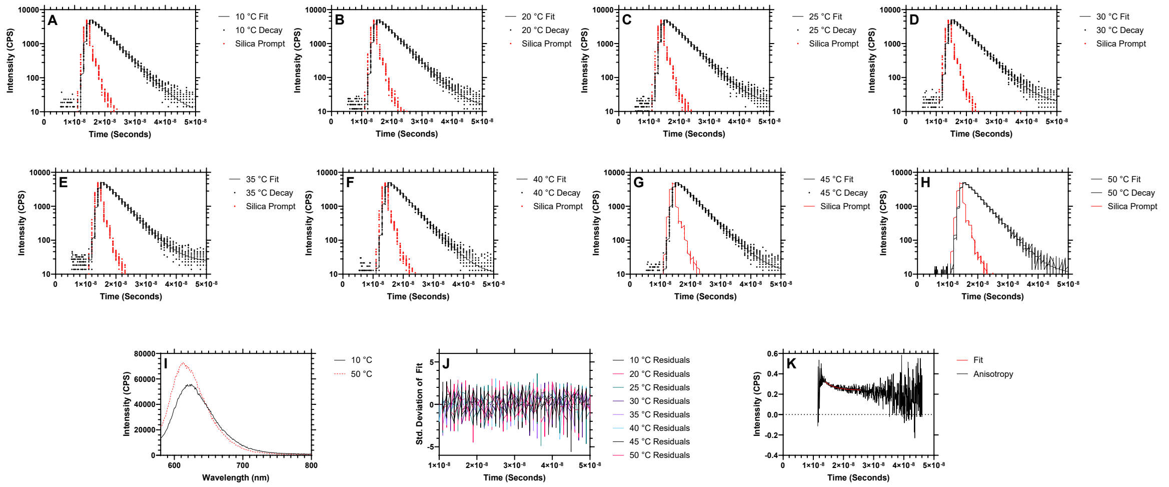

Figure ESI 20 – Fluorescent analysis of polymer **1** in water

A – H) Fluorescence lifetime emission at 640 nm following proton excitation fitted to a single exponential decay from 10 – 50 °C. **I)** Steady state emission at 10 and 50 °C. J) Residual standard deviations of fit of dual lifetime decays. K) Time resolved anisotropy (TRAMS) decay of polymer at room temperature.

This is raw data for Table 4

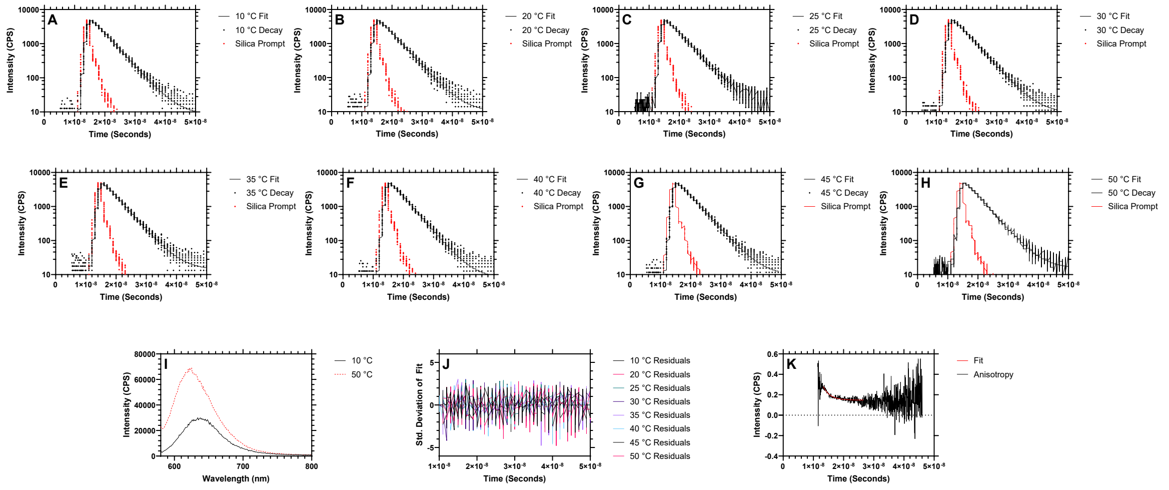

Figure ESI 21 – Fluorescent analysis of polymer **2** in water

A – H) Fluorescence lifetime emission at 640 nm following proton excitation fitted to a single exponential decay from 10 – 50 °C. **I)** Steady state emission at 10 and 50 °C. J) Residual standard deviations of fit of dual lifetime decays. K) Time resolved anisotropy (TRAMS) decay of polymer at room temperature.

This is raw data for Table 4

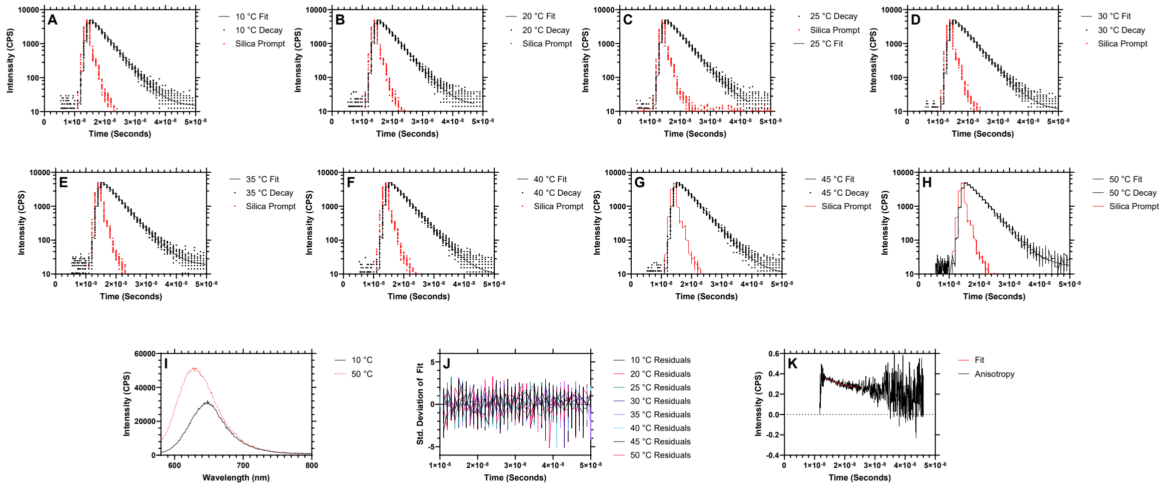

Figure ESI 22 – Fluorescent analysis of polymer **3** in water
A – H) Fluorescence lifetime emission at 640 nm following proton excitation fitted to a single exponential decay from 10 – 50 °C. **I)** Steady state emission at 10 and 50 °C. J) Residual standard deviations of fit of dual lifetime decays. K) Time resolved anisotropy (TRAMS) decay of polymer at room temperature.
This is raw data for Table 4

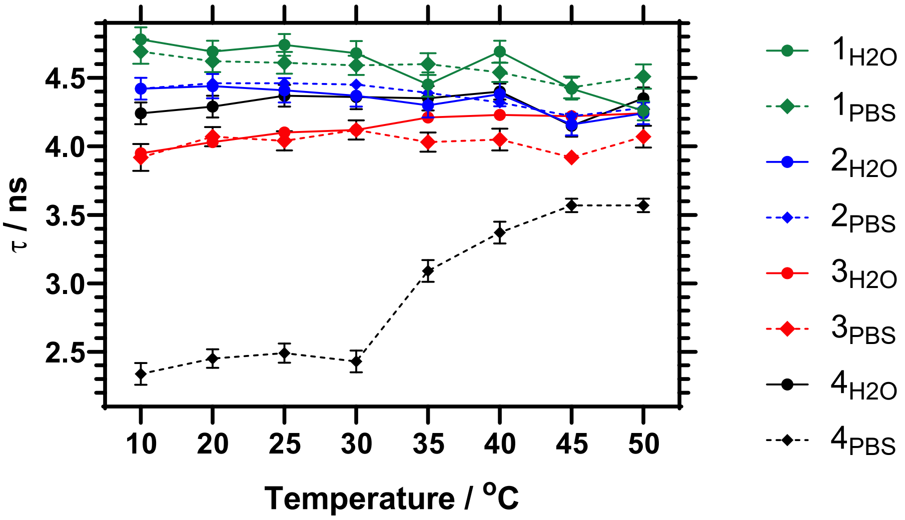

Figure ESI 23 – Fluorescent lifetime of polymers **1 – 4** in water and PBS (1 mg ml^{-1}).
This is raw data for Table 4

1.12 PNIPAM + Bacteria

Initially, a test was carried out to see whether polymer **2** is bactericidal. To do this *S. aureus* was spread on a brain heart infusion (BHI) agar plate so as to cover the entire plate with a lawn of bacteria. Three 10 µl drops of vancomycin (1 mg ml^{-1}) were dispensed on one side of the plate as a positive control for bacterial inhibition and three 10 µl drops of a polymer **2** solution (5 mg ml^{-1}) were added to the plate on the other side. The plate was then incubated at 37 °C overnight. Following overnight incubation, growth inhibition of *S. aureus* was clearly visible where the drops of vancomycin had been placed whereas there was no visible growth inhibition from the drops of polymer **2** (**Figure ESI 24a**). However, when the polymer was cultured in solution with bacteria, after 2 hours of polymer-bacteria incubation, it was found that the fluorescence intensity of the polymers increased quantitatively with the concentration of bacteria (**Figure ESI24 b-d**). When this data was interrogated it was found that at sufficient polymer concentration the response to *S. aureus* of all materials was significant, however the sensitivity of polymers **1-4** varied (**Table ESI 10**).

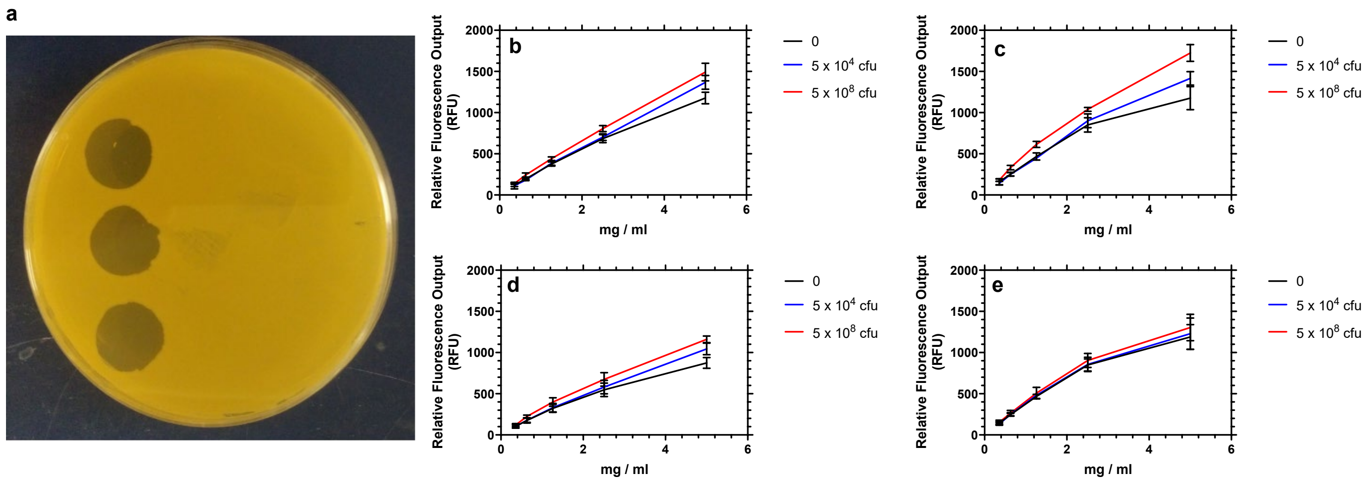

Figure ESI 24 – a) Comparison of bactericidal properties of vancomycin (left) and **2** (right) b-e) Increased polymer concentration shows quantitative fluorescence increase (λ_{ex} 639 nm, read at 32 °C) response to 4 hours bacterial incubation (0 – 1 x 10^8 cfu, 37 °C) of b) **1** c) **2**, d) **3** and e) **4**.
(*This is raw data of Figure 9 in main manuscript*)

Table ESI 10 - Evaluating the significance of the fluorescence difference between PNIPAM + *S. aureus* solutions

1 x 10^4 Cfu bacteria - low dosage

Conc / mg ml^{-1}	**1**	**2**	**3**	**4**
1.25	Ns	Ns	Ns	ns
2.5	Ns	Ns	Ns	Ns
5	***	***	**	Ns

1 x 10^8 Cfu bacteria - high dosage

Conc / mg ml^{-1}	**1**	**2**	**3**	**4**
1.25	Ns	*	Ns	Ns
2.5	*	**	*	**
5	****	****	****	****

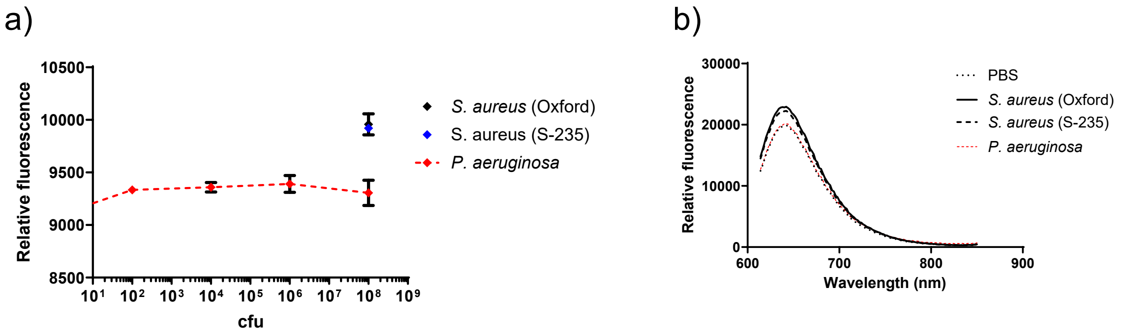

Figure ESI 25 – A) Incubation of polymer **2** with *P. aeruginosa* analysed via Plate Reader (λ_{em} 649 nm) *(same conditions as measurement with gram positive bacteria in Figure 9)*
B) Full Emission spectra of Polymer 2 with Bacteria in Fluoromax 4 Spectrometer

A study of polymer-bacteria agglutination (via a Matt Button assay) is shown in **Figure ESI 26**. A series was prepared with different polymer architectures, polymer concentrations and different bacterial concentrations. The data shows lower polymer concentrations (< 2.5 mg ml^{-1}) are not sufficient to prevent button formation, but at these concentrations it is still possible to see increased fluorescence intensity.

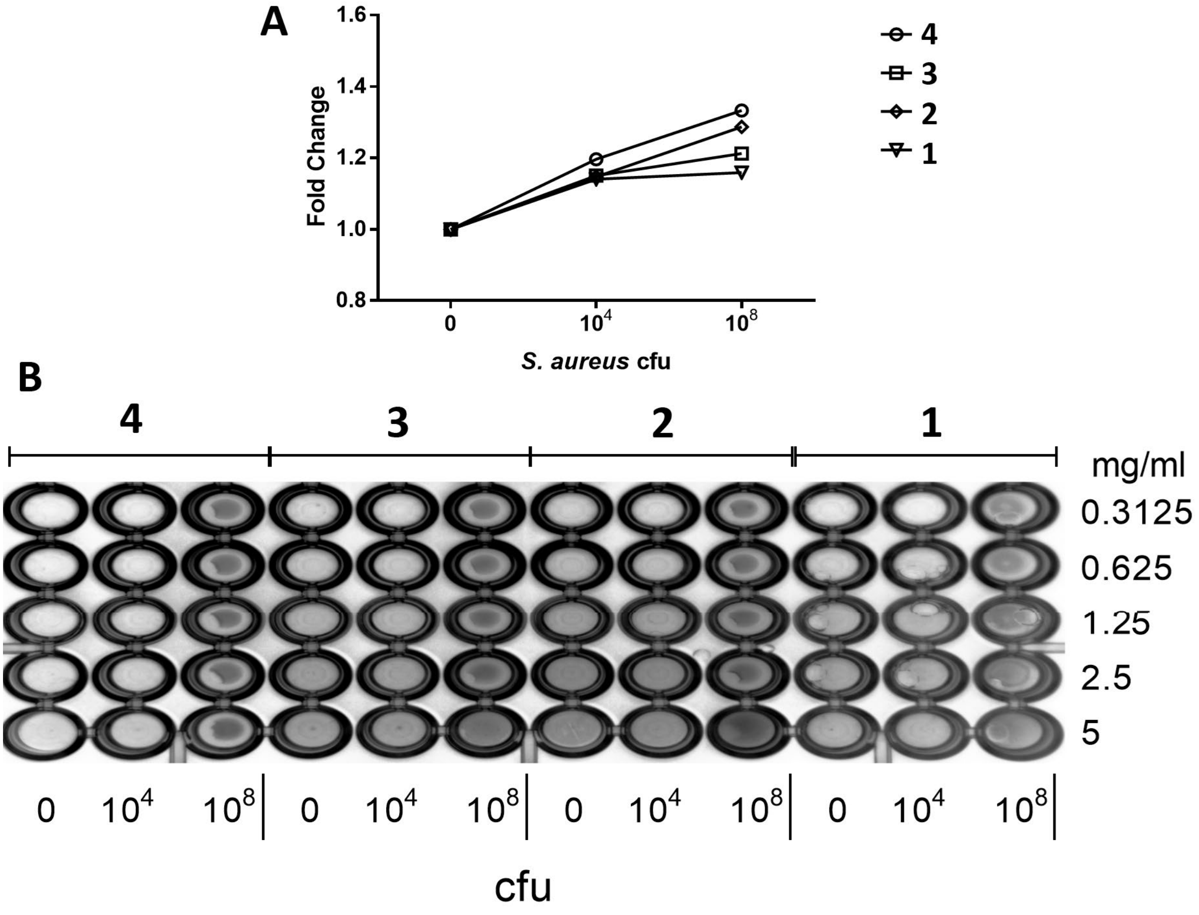

Figure ESI 26 – a) Fold change (I/I_0) of polymer emission within wells. b) full photograph of matt-button assay well expanded from Figure 2b, containing polymers **1-4** with different *S. aureus* concentrations.

Confocal analysis of aggregates from mixing bacteria with polymer **2** were analysed to show both the number of particles per field of view and the average size of these aggregates. Data is processed in ImageJ via threshold analysis to determine both number of particles and average particle size as shown in **ESI 27**. Data clearly showed that although the number of particles decrease enormously with increasing polymer concentration, the size of the aggregates increases in a far more linear fashion.

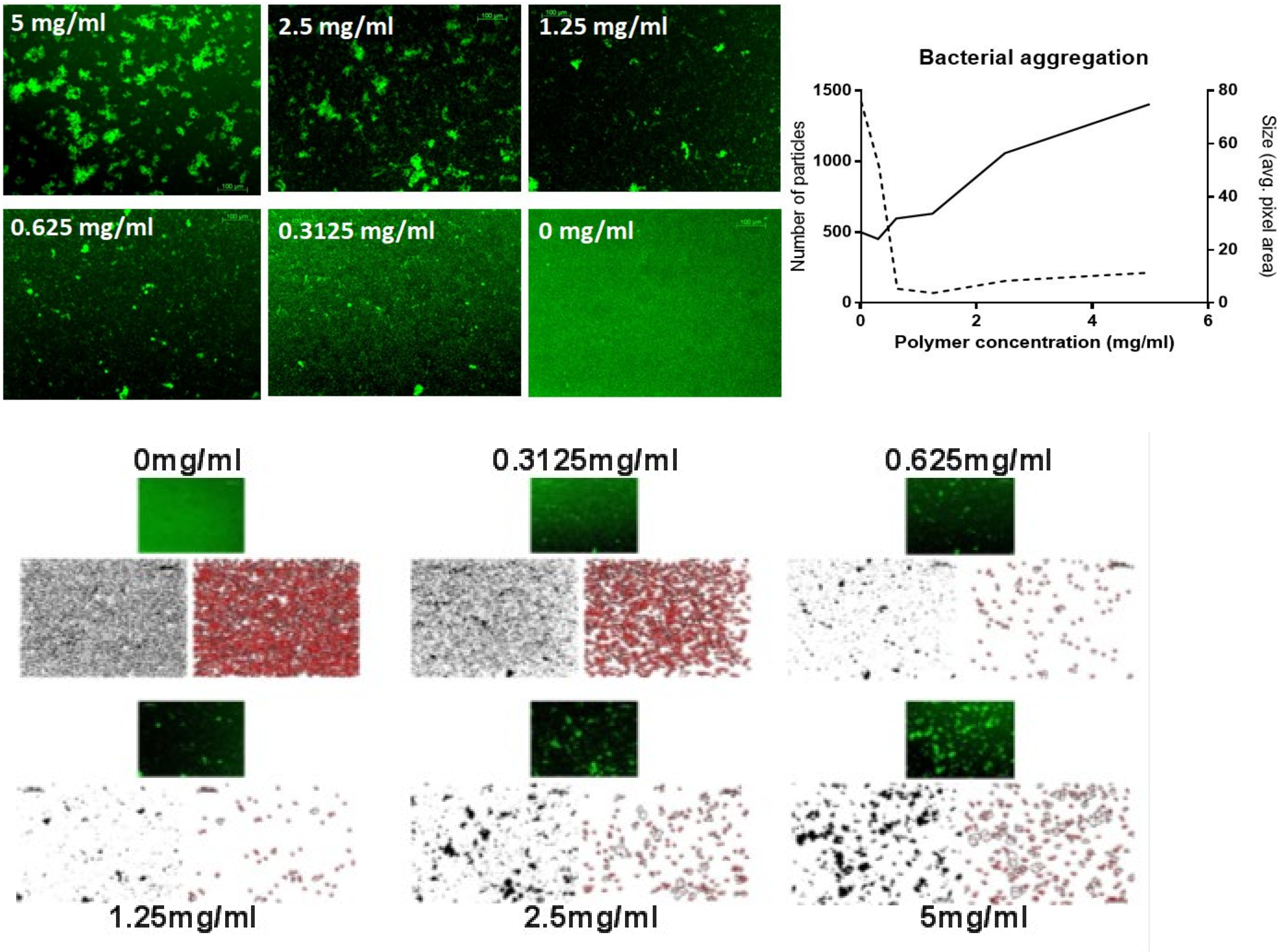

Figure ESI 27 – Confocal analysis of bacterial aggregation study, mixing varying concentrations of polymer **2** with pre-stained *S. aureus* at 37 °C. Data shows particle size increases with increased PNIPAM concentration. Fitting of particle size (avg. pixel area) carried out using ImageJ software.

Scanning Electron Microscopy

Examples of the polymer only and the bacteria only images are shown in Figure **ESI 28**, while polymer-bacteria interactions at various concentrations are shown in Figure 10 of the main manuscript.

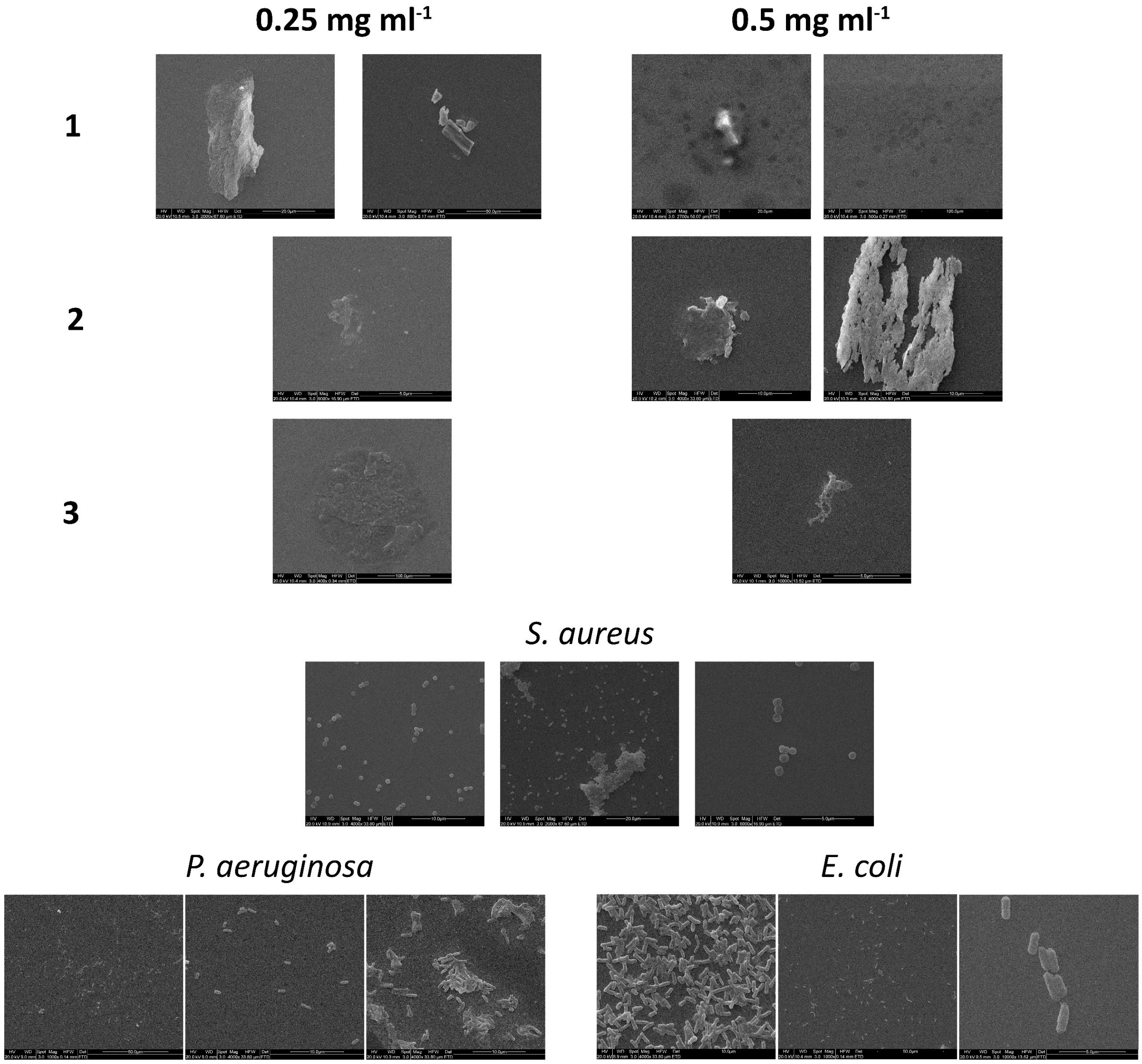

Figure ESI 28. – Control SEM analysis of polymer aggregates from dried dilute solutions on a glass slide. Bottom:) SEM images of 10^8 cfu *S. aureus, E. coli and P. aeruginosa* on glass plates in the absence of polymer.

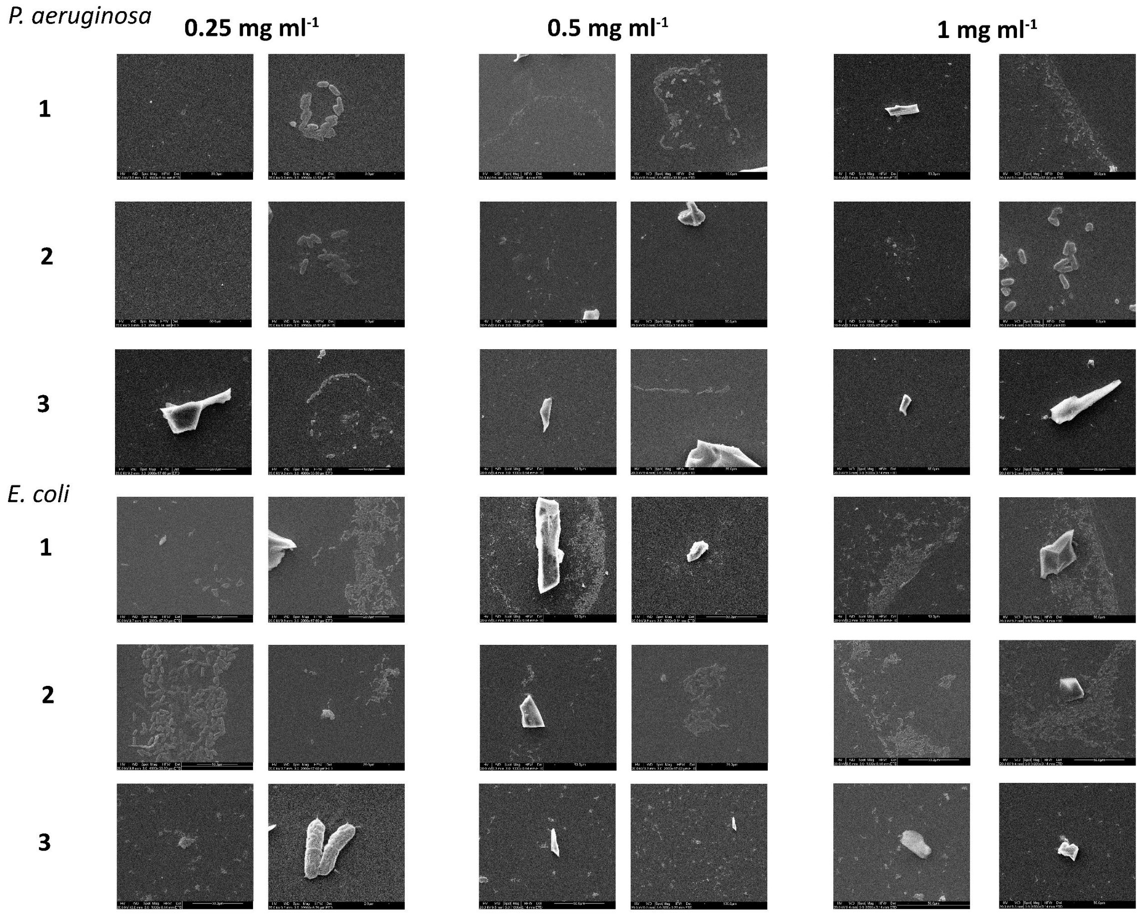

Figure ESI 29 – Negative control experiment showing no interaction of Polymers 1- 3 at 3 different concentrations, with *P. aeruginosa* and *E. coli*

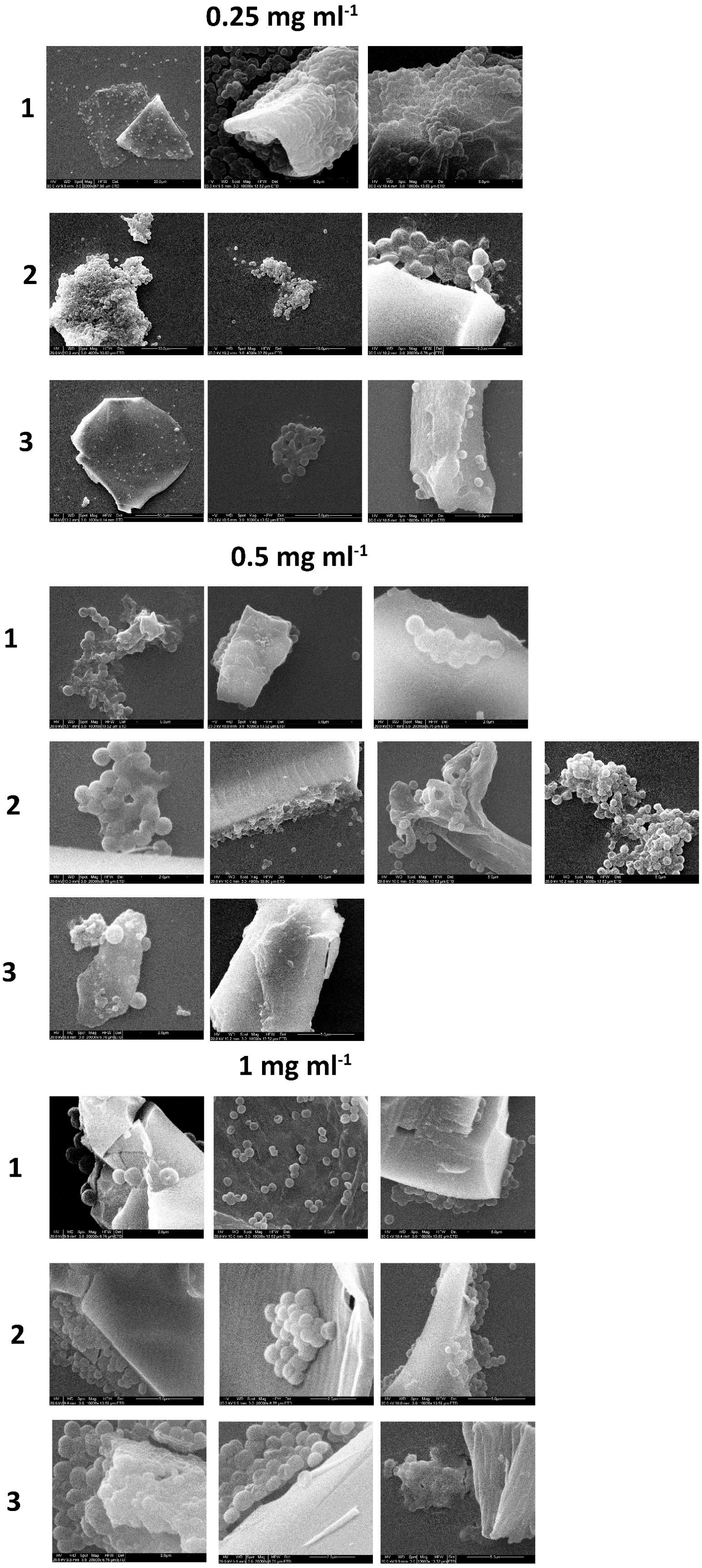

Figure ESI 30 – Further exemplar images of Figure 10.

2. ESI References

1 R. Plenderleith, T. Swift and S. Rimmer, Highly-branched poly(N-isopropyl acrylamide)s with core–shell morphology below the lower critical solution temperature, *RSC Adv.*, 2014, **4**, 50932–50937.

2 R. M. England and S. Rimmer, Hyper/highly-branched polymers by radical polymerisations, *Polym. Chem.*, 2010, **1**, 1533.

3 J. Shepherd, P. Sarker, K. Swindells, I. Douglas, S. MacNeil, L. Swanson and S. Rimmer, Binding bacteria to highly branched poly(N-isopropyl acrylamide) modified with vancomycin induces the coil-to-globule transition, *J. Am. Chem. Soc.*, 2010, **132**, 1736–1737.

4 T. Swift, M. Katsikogianni, R. Hoskins, P. Teratarantorn, I. Douglas, S. MacNeil, S. Rimmer, P. Teratanatorn, I. Douglas, S. MacNeil and S. Rimmer, Highly-Branched Poly(N-isopropyl acrylamide) Functionalised with Pendant Nile Red and Chain End Vancomycin for the Detection of Gram-Positive Bacteria, *Acta Biomater.*, 2019, **87**, 197–206.

5 T. Swift, R. Hoskins, R. Telford, R. Plenderleith, D. Pownall and S. Rimmer, Analysis using size exclusion chromatography of poly(N-isopropyl acrylamide) using methanol as an eluent, *J. Chromatogr. A*, 2017, **1508**, 16–23.

6 P. Teratanatorn, R. Hoskins, T. Swift, C. W. I. I. Douglas, J. Shepherd and S. Rimmer, Binding of Bacteria to Poly(N-isopropylacrylamide) Modified with Vancomycin: Comparison of Behavior of Linear and Highly Branched Polymers, *Biomacromolecules*, 2017, **18**, 2887–2899.

7 T. Swift, J. Lapworth, K. Swindells, L. Swanson and S. Rimmer, pH responsive highly branched poly(N-isopropylacrylamide) with trihistidine or acid chain ends, *RSC Adv.*, 2016, **6**, 71345–71350.

8 F. Neese, F. Wennmohs, U. Becker and C. Riplinger, The ORCA quantum chemistry program package, *J. Chem. Phys.*, 2020, **152**, 224108.

9 Y. Liu, J. Xu, L. Han, Q. Liu, Y. Yang, Z. Li, Z. Lu, H. Zhang, T. Guo and Q. Liu, Theoretical Research on Excited States: Ultraviolet and Fluorescence Spectra of Aromatic Amino Acids, *Interdiscip. Sci. Comput. Life Sci.*, 2020, **12**, 530–536.
